# Climate Change Projected to Double the Richness and Abundance of Soilborne Phytopathogenic Fungi in Southern Maritime Antarctica

**DOI:** 10.1111/gcb.70885

**Published:** 2026-05-12

**Authors:** Kevin K. Newsham, Elisabeth M. Biersma, Ian S. Acuña‐Rodríguez, Gabriel I. Ballesteros, Peter Convey, Marco A. Molina‐Montenegro, Izzy Newsham, Anders Priemé, Reti Ranniku, Cristian Torres‐Díaz, Gilda Varliero, William P. Goodall‐Copestake

**Affiliations:** ^1^ British Antarctic Survey Natural Environment Research Council Cambridge UK; ^2^ Natural History Museum of Denmark University of Copenhagen Copenhagen Denmark; ^3^ Centre for Integrative Ecology (CIE) Instituto de Ciencias Biológicas, Universidad de Talca Talca Chile; ^4^ Dirección de Investigación Vicerrectoría Académica, Universidad de Talca Talca Chile; ^5^ Department of Zoology University of Johannesburg Auckland Park South Africa; ^6^ School of Biosciences University of Birmingham Birmingham UK; ^7^ Millennium Institute, Biodiversity of Antarctic and Sub‐Antarctic Ecosystems (BASE) Santiago Chile; ^8^ Cape Horn International Center Puerto Williams Chile; ^9^ Centro de Investigación de Estudios Avanzados del Maule (CIEAM) Universidad Católica del Maule Talca Chile; ^10^ MRC Biostatistics Unit University of Cambridge Cambridge UK; ^11^ Section of Microbiology and Center for Volatile Interactions, Department of Biology University of Copenhagen Copenhagen Denmark; ^12^ Center for Permafrost, Department of Geosciences and Natural Resource Management University of Copenhagen Copenhagen Denmark; ^13^ Departamento de Ciencias Básicas Universidad del Bío‐Bío Chillán Chile; ^14^ Rhizosphere Processes Group Swiss Federal Research Institute WSL Birmensdorf Switzerland; ^15^ Royal Botanic Garden Edinburgh Edinburgh UK

**Keywords:** air temperature, animal and plant pathogenic fungi, DNA metabarcoding, least absolute shrinkage and selection operator (LASSO) regression, precipitation, shared socioeconomic pathways, soil pH value

## Abstract

How climate change impacts pathogens in the natural environment is a critical ecological question. Yet, little is known of how rapid ongoing climate change in Antarctica and Patagonia will influence the fungal pathogens in the barren soils typical of these regions. Here, using DNA metabarcoding and LASSO regression, we identify climatic factors—and notably mean annual air temperature—as the best predictors for the taxonomic richness and relative abundance of fungal pathogens of plants and animals in barren soils sampled from along a 1900‐km transect through sub‐ and Maritime Antarctica and Patagonia. Projected changes to climate under three shared socioeconomic pathway scenarios (SSP1‐2.6, SSP3‐7.0, and SSP5‐8.5) were used to predict how soilborne pathogenic fungal communities will alter by 2071–2100. The SSP3‐7.0 and SSP5‐8.5 scenarios were projected to cause approximate doublings to the richness and abundance of phytopathogenic fungi in southern Maritime Antarctic soils. Weaker effects of these two scenarios were found on phytopathogens elsewhere on the transect and on animal pathogenic fungi. Changes to climate under the SSP1‐2.6 scenario had negligible impacts on both guilds. Edaphic factors explained lower amounts of variance in soil pathogenic fungal diversity and relative abundance than climatic factors. Our findings indicate higher frequencies of fungi causing grey molds, stem necroses, blights, scabs and leaf spots in warmer soils. They foreshadow end‐of‐century increases in the richness and abundance of phytopathogenic fungi inhabiting the barren soils of southern Maritime Antarctica, posing a threat to the region's flora as it colonizes these soils.

## Graphical abstract

We sequenced fungal DNA in barren soils sampled from 12 sites along a 1900‐km transect through sub‐ and Maritime Antarctica and Patagonia. Using LASSO regression, we found air temperature ‐ and notably mean annual air temperature (BIO1) ‐ to be a highly significant predictor for the richness and relative abundance of soilborne plant pathogens. Our analyses predict approximate end‐of‐century doublings to the richness and abundance of phytopathogenic fungi in southern Maritime Antarctic soils under two socioeconomic pathway scenarios (SSP3‐7.0 and SSP5‐8.5), posing a threat to the region's flora as it colonizes these soils.

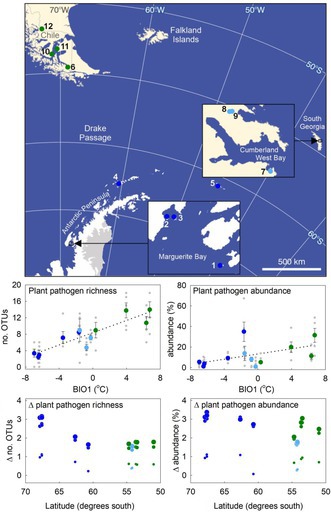

## 1 Introduction

Soil is inhabited by a wide range of fungal pathogens that have profound impacts on ecosystems, agriculture, and human health (Fisher et al. 2012; Singh et al. 2023; World Health Organization 2022). Over the previous decade, it has become evident that the taxonomic diversity and abundance of soilborne pathogenic fungi are strongly influenced by air temperature and precipitation (Delgado‐Baquerizo et al. 2020; Mikryukov et al. 2023; Tedersoo et al. 2014; Větrovský et al. 2019), with changes to the Earth's climate since 1960 having led to a ~7‐km year^−1^ shift of these microbes towards the poles (Bebber et al. 2013). Such shifts, which have allowed the establishment of fungal diseases in hitherto unsuitable habitats, are facilitated by the efficient airborne dispersal of fungal spores (Abrego et al. 2024), with fungi consequently being the most widespread and rapidly dispersing group of plant pathogens and pests worldwide (Bebber et al. 2014). Although rising air temperature and precipitation are thought to be the main factors causing fungal pathogen range shifts (Li et al. 2023; Větrovský et al. 2019), human‐mediated dispersal, vegetation cover, and edaphic factors also influence the distribution of soilborne fungi (Delgado‐Baquerizo et al. 2020; Mahon et al. 2024; Newsham et al. 2021). Furthermore, increased host susceptibility to fungal infection and the accumulation of transposons in fungal genomes at higher temperatures may facilitate the emergence of previously unknown thermotolerant fungi (Garcia‐Solache and Casadevall 2010) and rapid fungal microevolution (Gusa et al. 2023).

Fungal pathogens cause ecologically and economically important diseases of both plants and animals. In the natural environment, disease outbreaks caused by aggressive phytopathogens assigned to genera such as *Cryphonectria* and *Ophiostoma* have been responsible for the near extirpation of several plant species (Agrios 2009; Harvell et al. 2002), and in agricultural settings, infections by phytopathogenic fungi such as *Fusarium* and *Botrytis* lead to crop yield losses each year sufficient to feed 8.5% of the world's population, threatening food security (Bebber et al. 2013; Delgado‐Baquerizo et al. 2020; Fisher et al. 2012; Singh et al. 2023). Furthermore, whilst viruses and bacteria were formerly regarded as the main etiological agents of animal disease, this perception has changed over the last three decades following the emergence of devastating fungal diseases, notably chytridiomycosis of amphibians and white nose syndrome of bats, caused by species of *Batrachochytrium* and *Pseudogymnoascus*, respectively (Fones et al. 2017). In humans, following the widespread immunosuppression that occurred in the wake of the HIV‐AIDS pandemic, infections caused by critical fungal pathogens such as yeasts assigned to *Candida* and *Cryptococcus* now cause 1.5 million fatalities each year (Rodrigues and Nosanchuk 2023). Understanding how climate change will impact the diversity and abundance of fungal pathogens (Harvell et al. 2002) is thus central to tackling the sharp increases in the global occurrence of fungal diseases of plants and animals that have been recorded since the turn of the millennium (Fisher et al. 2012).

Previous studies showing climate to constrain the diversity and abundance of soilborne fungal pathogens have typically excluded Antarctic soils and have included relatively few data for Patagonian soils (Delgado‐Baquerizo et al. 2020; Tedersoo et al. 2014; Větrovský et al. 2019). Although the global phytopathogen assessment of Li et al. (2023) did include soils from Antarctica, its main focus was on forest and cropland soils, diminishing its predictive power for the fungal communities of barren soils. These soils, which are inhabited by markedly different taxa of fungi than those inhabiting densely vegetated soils on other continents (Newsham et al. 2021), are frequent at high latitudes. Moreover, projections for how soilborne animal pathogenic fungi in Antarctica will respond to climate change are absent from the literature. These gaps in current knowledge are significant, given the need to preserve the pristine ecosystems of Antarctica in the face of rapid ongoing climate change (Bracegirdle et al. 2020) and pressure from national research operators and the tourism industry on the continent, both of which increase the likelihood of its invasion by novel pathogens (Convey and Peck 2019). Here, in a study of barren sub‐ and Maritime Antarctic and Patagonian soils, we determine which of 13 climatic and edaphic factors best explain the taxonomic richness and relative abundance of fungi assigned to genera that cause, or are associated with, diseases of plants or animals. By estimating end‐of‐century changes to air temperature and precipitation under three shared socioeconomic pathway (SSP) scenarios (Riahi et al. 2017), we show that climate change is likely to approximately double the present‐day richness and abundance of phytopathogenic fungi in the barren soils of southern Maritime Antarctica, posing a threat to the region's flora as it expands into these soils.

## 2 Materials and Methods

### 2.1 Sampling

**FIGURE 1:**
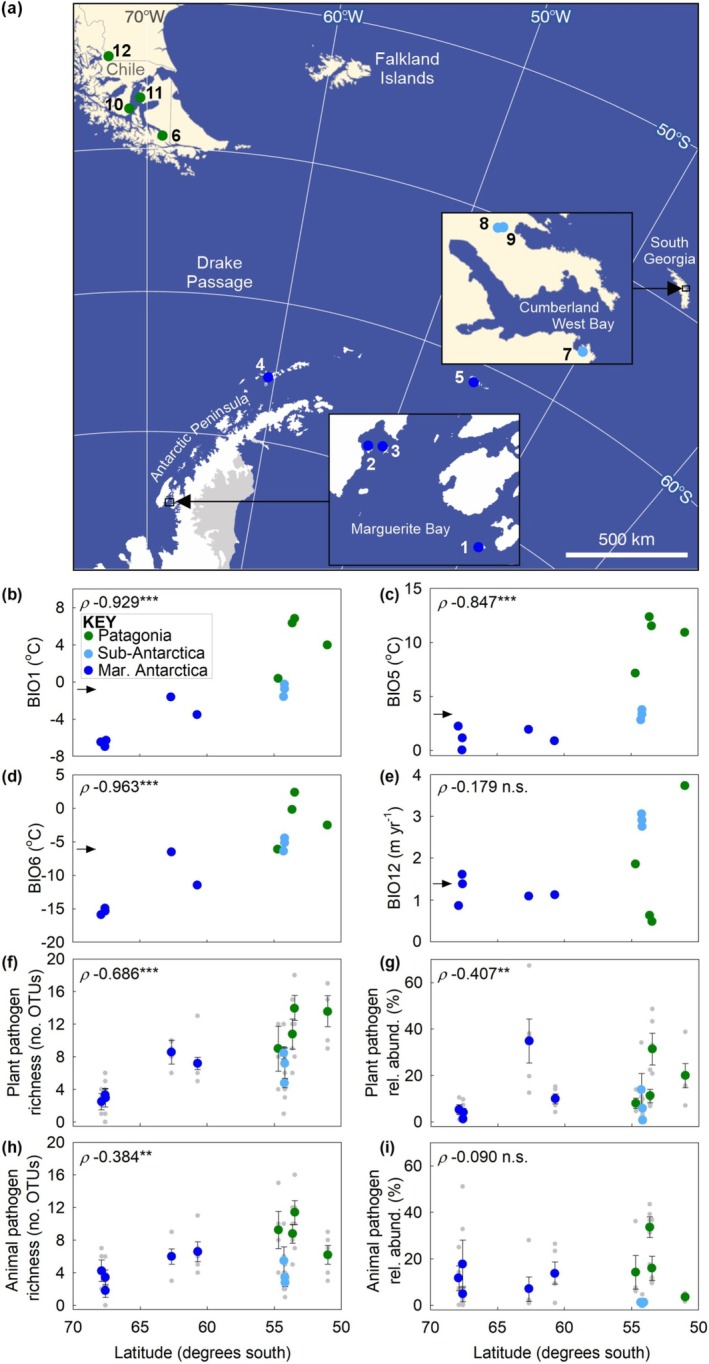
Sampling sites, and climatic factors, OTU richness and relative abundance of soilborne fungal pathogens as functions of latitude. (a) Locations of the 12 sampling sites from which soils were collected. (b–e) Mean annual air temperature (BIO1), maximum air temperature of the warmest month (BIO5), minimum air temperature of the coldest month (BIO6) and mean annual precipitation (BIO12) as functions of latitude. (f–i) The OTU richness and relative abundance of plant and animal pathogenic genera as functions of latitude. Note that the sampling sites are color‐coded for geographical region (see key in panel b) and that the *x*‐axes of panels b–i are identically scaled. Spearman's rank correlation coefficients (*ρ*) are shown in panels b–i, with asterisks denoting statistical significance (**p < 0.01 and ***p < 0.001). Map lines delineate study areas and do not necessarily depict accepted national boundaries. The sampling sites were located at: (1) Lagotellerie Island (67.886°S, 68.420°W); (2) Léonie Island (67.602°S, 68.346°W); (3) Lagoon Island (67.593°S, 68.234°W); (4) Hannah Point, Livingston Island (62.654°S, 60.610°W); (5) Backslope, Signy Island (60.710°S, 45.592°W); (6) Karukinka Natural Park, Chile (54.688°S, 68.978°W); (7) Maiviken, Thatcher Peninsula, South Georgia (54.288°S, 36.495°W); (8) Upper Husdal Valley, Busen region, South Georgia (54.196°S, 36.740°W); (9) Pohlia Falls, Busen region, South Georgia (54.191°S, 36.729°W); (10) Fuerte Bulnes, Chile (53.633°S, 70.916°W); (11) Porvenir, Chile (53.469°S, 70.252°W); and (12) Torres del Paine, Chile (51.015°S, 73.060°W). Abbreviations: Mar., Maritime; n.s., not significant; OTUs, operational taxonomic units; rel. abund., relative abundance.

Fifty‐seven barren soil samples were collected in January–February 2018 from 12 sites across a 1900‐km‐long transect between Lagotellerie Island in Maritime Antarctica at 67.9°S and Torres Del Paine in Patagonian Chile at 51.0°S (Figure 1a). At each site, 4–5 soil samples (each *c*. 20 g fwt) were collected 1–2 m distant from sparse individuals of Antarctic pearlwort (*Colobanthus quitensis*), one of the two vascular plant species that occur along the transect. The soils, which were sampled at least 2‐m apart from each other, were collected into sterile containers that were stored at *c*. 4°C for several hours before being frozen at −20°C, prior to transfer to the laboratory at the same temperature.

### 2.2 DNA Isolation and Next Generation Sequencing

The soil samples were defrosted, mixed thoroughly and freeze‐dried. DNA was isolated from subsamples (0.25 g) using a FastDNA Spin Kit for Soil (MP Biomedicals, Solon, Ohio, USA) following the manufacturer's protocol. The internal transcribed spacer (ITS) 2 region of fungal DNA was amplified using the primers ITS4 and ITS7 (Ihrmark et al. 2012; White et al. 1990). Polymerase chain reaction (PCR) amplifications consisted of two steps. In the first, primers and template DNA (1 μL) were added to Illustra puReTaq Ready‐To‐Go PCR Beads (GE Healthcare, Little Chalfont, UK) and were subjected to thermocycling in 20‐μL reactions at 94°C for 2 min, then 35 cycles at 94°C for 30 s, 56°C for 30 s and 72°C for 30 s, followed by extension at 72°C for 5 min. In the second step, 2 μL of the 10× diluted PCR product was added to a reaction mixture consisting of 0.15 μL DNA polymerase (AccuPrime Taq DNA Polymerase High Fidelity, Invitrogen, Thermo Fisher Scientific, MA, USA), 2 μL 10× AccuPrime PCR Buffer II, 1 μL (770 nM) of each custom‐tagged forward and reverse primer and 13.85 μL sterile water in a 20‐μL reaction volume. The reaction mixtures were subjected to thermocycling at 94°C for 2 min, then 14 cycles at 94°C for 30 s, 56°C for 30 s and 72°C for 30 s, followed by extension at 72°C for 5 min. The amplicons were then purified by electrophoresis in a 1% agarose gel followed by gel extraction (Montage Gel Extraction Kit, Millipore, Billerica, MA, USA). Amplicon concentrations were quantified for all samples using Qubit dsDNA HS assays and fluorescence was measured using a Qubit fluorometer (Invitrogen). Samples were pooled in equimolar amounts and paired‐end sequenced over two MiSeq flowcells (Illumina, San Diego, California, USA).

### 2.3 Sequence Processing

Demultiplexed sequences from each of the samples were trimmed of amplification primers using cutadapt 1.18 (Martin 2011). The DADA2 package v. 1.22.0 (Callahan et al. 2016) was subsequently used in R v. 4.1.1 (R Core Team 2022) for quality filtering, inference of amplicon sequence variants (ASVs, i.e., DNA sequences representing the genetic diversity present in the original sample) and taxon assignment. Quality filtering removed all sequences with ambiguous bases (trncQ = 11), > 2 expected errors and lengths < 50 bp. Error rates were estimated for forward and reverse sequences and ASVs were inferred before merging with a minimum overlap of 30 bp. Putative chimeras were removed using the consensus method. Taxonomies were assigned to the ASVs using the RDP classifier (Wang et al. 2007) with the sh_general_release‐dynamimc_all_25.07.2023 reference from UNITE (Abarenkov et al. 2023). The decontam v. 1.14.0 package (Davis et al. 2017) was then used in R to remove putative contaminants identified in negative control amplification samples using a threshold value of 1.0. All putative contaminants were cross checked through manual inspection.

### 2.4 Taxon Inference

Operational taxonomic units (OTUs, i.e., groups of DNA sequences similar to each other at approximately species level) were inferred to facilitate comparisons with previous studies on the richness of soil pathogenic fungi (e.g., Mikryukov et al. 2023; Tedersoo et al. 2014). In order to infer OTUs, the taxonomy‐assigned, decontaminated ASVs were clustered at 98% similarity over 90% of sequence length (Tedersoo et al. 2021) using the BLASTCLUST algorithm in the blast‐legacy v. 2.2.26 package (Camacho et al. 2009). One hundred and sixty one of the OTUs were singletons, with 51 OTUs comprising more than one ASV. The consensus taxonomies for these multi‐ASV OTUs were all unambiguously defined, with the constituent ASVs in each OTU being in agreement down to genus level. The majority of the diversity analyses reported here were focused on OTU richness. However, the LASSO and univariate regression analyses described in section 2.8 were also repeated on ASV richness (genetic diversity) to assess if diversity patterns derived from analyses of unclustered ASVs yielded the same results as those based on OTU richness (species diversity).

### 2.5 Assignments of Taxa to Guilds

Fewer than half of the OTUs could be assigned to species, and hence, as in previous studies (e.g., Delgado‐Baquerizo et al. 2020; Li et al. 2023), assignments of taxa to the plant or animal pathogenic fungal guilds were made at the genus level. Preliminary analyses using the FUNguild database (Nguyen et al. 2016) showed that few genera could be assigned with highly probable confidence rankings to each guild. Manual searches were hence made in Web of Science (https://www.webofscience.com/) using all genus names and “pathogen*” or “disease” as terms, with a genus being assigned to a guild when there was evidence from the literature that at least one of its members causes, or is associated with, diseases of plants or animals. Virulence, which is dependent on a wide range of factors including host physiology and environment (Casadevall and Pirofski 2001), was not taken into account when assigning genera to each guild. The 105 genera assigned to the plant or animal pathogen guilds, along with supporting literature, are listed in Table S1. Eight genera, including *Alternaria, Cladosporium*, and *Fusarium*, were assigned to both guilds.

### 2.6 Edaphic Factors

Soil pH value was measured in a suspension of 5 g fresh soil and 20 mL of double‐distilled water. Soil C/N ratio was calculated following measurement of total C and N in sub‐samples of freeze‐dried soil using a TruSpec CN analyser (Leco, St Joseph, MI, USA). Water‐soluble C and N were extracted from fresh soil (5 g) in 25 mL of double‐distilled water for 1 h. Following filtration through Whatman GF‐D filter paper, concentrations of total dissolved organic C (TDOC) were measured using a Shimadzu TOC‐L CSH/CSN total organic C analyzer (Shimadzu, Kyoto, Japan). Total dissolved N (TDN) was measured with a FIAstar 5000 flow injection analyser (Foss Tecator, Höganäs, Sweden) after digesting extracts in H_2_SO_4_ with Se as a catalyst. Concentrations of NO_3_^−^‐N and NH_4_^+^‐N in solution were also measured using a FIAstar 5000 flow injection analyser using cadmium reduction and the indophenol blue and molybdenum blue methods, respectively.

### 2.7 Climatic Factors

Seven climatic predictors for each site (Table S2), based on a 30‐arc sec resolution for 1981–2010, were extracted from the CHELSA database v. 2.1 (Karger et al. 2017) using the terra v. 1.6–47 package (Hijmans et al. 2022) in the R environment v. 4.1.3. Although we currently lack an independent, gauge‐corrected reference precipitation dataset for the transect, the CHELSA database provides a physically‐based, bias‐corrected climatology that performs well globally, represents one of the few climatic datasets available for Antarctica and gives close fits (*r* ^2^ > 0.9) to precipitation data recorded at Maritime Antarctic stations (Karger et al. 2017). Given the scarcity and uncertainty of local gauge data—which underestimate snowfall owing to wind‐induced undercatch (Turner et al. 2002)—we consider the database to be a reasonable choice for describing spatial precipitation gradients in the current study.

Projected mean annual air temperature (BIO1), maximum air temperature of the warmest month (BIO5), minimum air temperature of the coldest month (BIO6) and mean annual precipitation (BIO12) values for each site in 2071–2100 were extracted from the CHELSA‐BIOCLIM+ database (Brun et al. 2022). These projected values, which were again based on a 30‐arc sec resolution, used ensemble predictions derived from five Earth system models (gfdl‐esm4, ipsl‐cm6a‐lr, mpi‐esm1‐2‐h, mri‐esm2‐0, and ukesm1‐0‐ll) for three shared socioeconomic pathway (SSP) scenarios (SSP1‐2.6, SSP3‐7.0, and SSP5‐8.5). These scenarios assume low, high, and very high greenhouse gas emissions to the atmosphere, respectively, with emissions varying between scenarios depending on socio‐economic assumptions, levels of climate change mitigation and, for aerosols and non‐methane ozone precursors, air pollution controls (IPCC 2021; Riahi et al. 2017).

### 2.8 Statistical Analyses

Non‐parametric Spearman's rank correlations were initially used to assess the relationships between latitude and each climatic or edaphic factor. Least absolute shrinkage and selection operator (LASSO) regression, a form of multiple regression incorporating regularisation (Tibshirani 1996), was then implemented in R using the glmnet v. 4.1–8 package (Friedman et al. 2010) with Gaussian family and default parameter values to determine the factors best explaining the richness (expressed as the number of OTUs or ASVs) and the relative abundance (expressed as the percentage abundance in the entire soil fungal community) of each guild. LASSO regression was chosen for these analyses because of the multicollinearity in the climatic and edaphic factor data (Figure S1) and because regularisation limits model capacity, reducing the risk of overfitting associated with, for example, stepwise regression (McNeish 2015). It uses L1 regularisation, which encourages some coefficients to be exactly zero, effectively performing variable selection. The degree of regularisation (lambda) was adjusted so that four predictor variables were selected in each model, with the four‐factor models with the lowest Akaike information criterion, indicative of satisfactory fit and model simplicity, being selected for the OTU or ASV richness and relative abundance of each guild. ANOVA using default parameters was applied to determine the significance of each of the four predictors, and linear or quadratic univariate regression models were applied to the data in order to determine regression coefficients. Relative abundance data were Hellinger‐transformed prior to analyses and data were rarefied to 5918 sequences, the minimum number of DNA reads per sample (Table S3), with the iNEXT package (Chao et al. 2014, 2016). The 2–3 samples from which plant or animal pathogenic fungal genera were absent were excluded from these analyses. Soils from the same sites were assumed to have been exposed to the same temperature and precipitation.

The intercepts and coefficients derived from LASSO regression were then used to calculate the mean predicted OTU richness and relative abundance of the guilds at each site in 2071–2100 using the projected BIO1, BIO5, BIO6, and BIO12 values extracted from the CHELSA‐BIOCLIM+ database. Since there is previous evidence for a negative association between the C/N ratio of barren soils sampled from 60° to 72°S and air temperature (Newsham et al. 2016), mean C/N ratio at each site in 2071–2100 was calculated based on a 0.169 unit reduction in this factor for each degree Celsius increase in BIO5 (Figure S2) and was included as a predictor in the calculations for the relative abundance of animal pathogenic fungi. In contrast, Spearman's rank correlations indicated no relationships between soil pH value and any measures of air temperature or precipitation (Figure S1), corroborating previous observations showing that soil pH value does not alter along 1650–2200‐km transects through sub‐ and Maritime Antarctica (Horrocks et al. 2020; Newsham et al. 2016), and those showing the pH value of barren Maritime Antarctic soil to be unaffected by 5 years of warming with open top chambers (Figure S3). Soil pH value was hence included, but was held constant, in the calculations for the relative abundance of plant pathogenic fungi and the OTU richness of both guilds. Similarly, NH_4_^+^‐N concentration was included, but was held constant, in the calculations for the OTU richness of plant pathogenic fungi and the relative abundance of animal pathogenic fungi, since there is robust evidence that increased concentrations of inorganic nitrogen in soils at the three southernmost sites studied here are caused not by low air temperatures but by the proximity of the sites to seal or seabird colonies (Bokhorst et al. 2019).

Principal component analysis (PCA) in MINITAB v. 22 was used to determine the associations between climatic and edaphic factors and the Hellinger‐transformed relative abundance of each fungal genus in the entire soil fungal community. Indicator analysis using the indicspecies v. 1.7.15 package in R and the multipatt() function (De Cáceres and Legendre 2009) was used to determine the genera of plant and animal pathogenic fungi representative of soils from sites with values for climatic and edaphic variables less than and greater than the median value for the transect (Figures 1b–e and S4; Table S2). The dependent variable in these analyses was the percentage relative abundance of each genus in the entire soil fungal community, and the independent climatic and edaphic variables were restricted to those selected as predictors by LASSO regression. In order to reduce type I errors in the analyses, *p* values were adjusted with Benjamini–Hochberg correction (Benjamini and Hochberg 1995).

## 3 Results

### 3.1 Current Climate and Soilborne Fungal Pathogen OTU Richness and Abundance

Spearman's rank correlations indicated that current air temperatures derived from the CHELSA dataset increased northwards along the transect, with BIO1 rising from between −6.4°C and −7.0°C at sites 1–3 in southern Maritime Antarctica to 4.0°C–6.8°C at sites 10–12 in Patagonia (Figure 1b). BIO5 similarly rose from 0.1°C to 2.3°C at sites 1–3 to 11.0°C–12.4°C at sites 10–12 (Figure 1c). BIO6 also increased from between −15.1°C and −16.0°C at sites 1–3 to between −2.6°C and 2.5°C at sites 10–12 (Figure 1d). BIO12 did not alter along the transect owing to anomalously low precipitation at sites 10 and 11 in Patagonia (0.48–0.63 m year^−1^; Figure 1e). Mean air temperature seasonality (BIO4), annual range in air temperature (BIO7), and precipitation seasonality (BIO15) each declined between the southernmost and northernmost points of the transect (Figure S4a–c). For the edaphic factors, although mean soil pH value did not change along the transect, mean C/N ratio, and the concentrations of NH_4_^+^‐N, NO_3_^−^‐N, total dissolved nitrogen, and total dissolved organic carbon each declined between 67.9°S and 51.0°S (Figure S4d–i).

Spearman's rank correlations also indicated that the current OTU richness of plant pathogenic genera increased significantly between 67.9°S and 51.0°S (Figure 1f). The mean richness of these genera was lowest at sites 1–3 in southern Maritime Antarctica (2.5–3.4 OTUs), was moderate at sites 4 and 5, the more northerly Maritime Antarctic sites (7.2–8.6 OTUs), and at sites 7–9 on sub‐Antarctic South Georgia (4.8–8.5 OTUs), and was highest at sites 6 and 10–12 in Patagonia (9.0–14.0 OTUs; Figure 1f). Similarly, the relative abundance of plant pathogenic fungi increased northwards along the transect, with low abundances of these genera at sites 1–3 (1.1%–5.3%) and sites 7–9 (1.0%–13.8%), and higher abundances at sites 4 and 5 (10.0%–35.0%) and sites 6 and 10–12 (8.2%–31.4%; Figure 1g). As for the OTU richness of plant pathogens, that of animal pathogenic fungi also increased northwards along the transect (Figure 1h). The mean richness of these genera, which was lowest at sites 1–3 (1.8–4.3 OTUs) and sites 7–9 (2.8–5.5 OTUs), was higher at sites 4 and 5 (6.0–6.6 OTUs) and sites 6 and 10–12 (6.2–11.4 OTUs; Figure 1h). In contrast, the relative abundance of animal pathogens was not associated with latitude, with the lowest abundances of these genera being recorded at sites 7–9 (0.6%–1.4%), and similar abundances being found at sites 1–6 and site 12 (3.5%–17.8%; Figure 1i). Two of the three highest relative abundances of animal pathogenic fungi were recorded at sites 10 and 11 in Patagonia (16.0% and 33.6%, respectively; Figure 1i).

### 3.2 Factors Predicting Soilborne Fungal Pathogen Richness and Abundance

**FIGURE 2:**
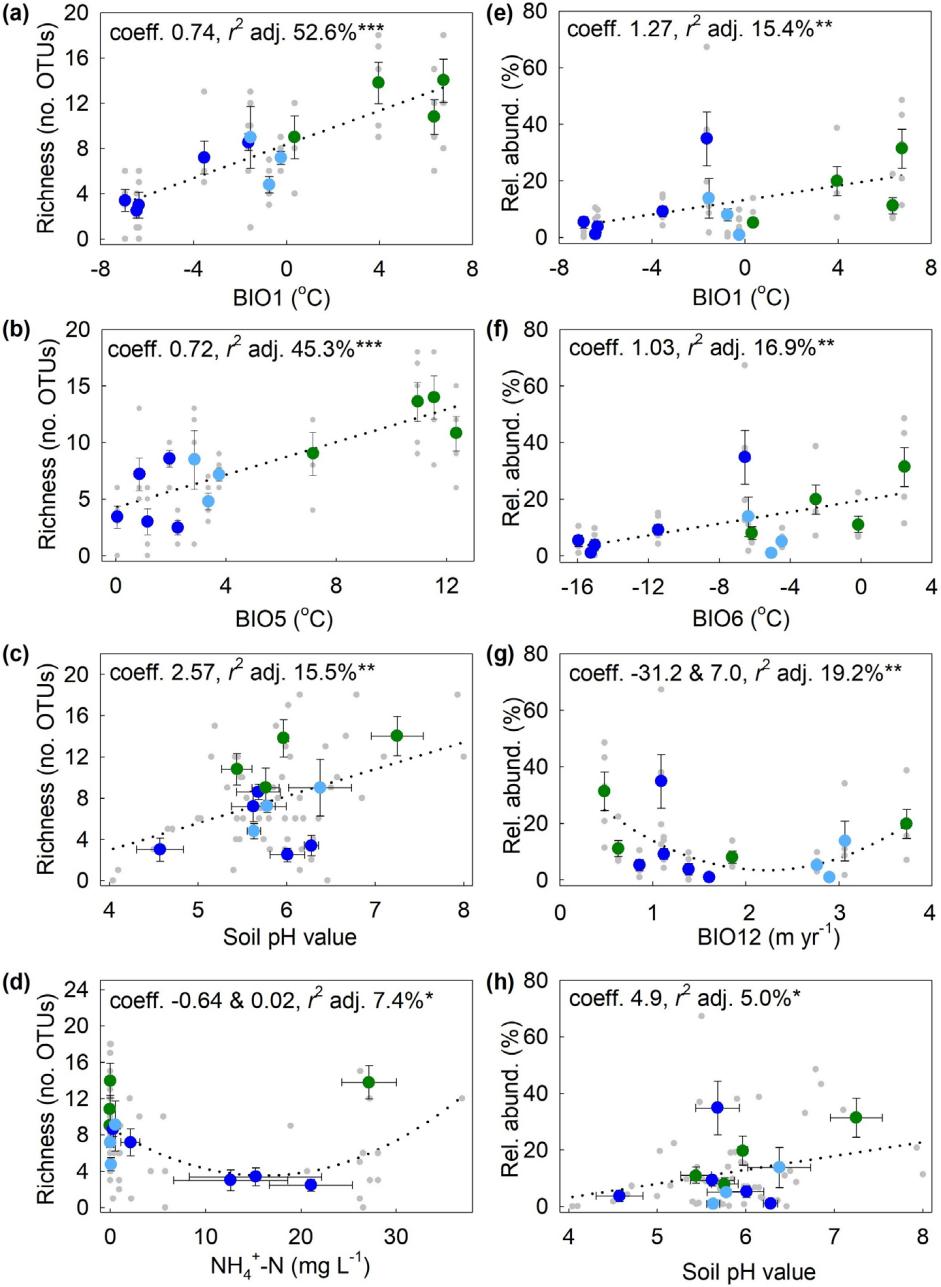
The OTU richness and relative abundance of soilborne plant pathogenic fungi as functions of climatic and edaphic factors selected by LASSO regression. (a–d) OTU richness as functions of BIO1, BIO5, soil pH value, and NH_4_^+^‐N concentration. (e–h) Relative abundance as functions of BIO1, BIO6, BIO12, and soil pH value. Values are means ± SEM and grey dots show individual data points. Dotted lines show linear or quadratic fits from regression models and asterisks denote statistical significance (*p < 0.05, **p < 0.01 and ***p < 0.001). The first and second coefficients shown in panels d and g are linear and squared components, respectively. Sampling sites are color‐coded for geographical region (see key in Figure 1b). Abbreviations: adj., adjusted; Coeff., coefficient. Other abbreviations and notation as in Figure 1.

Residual plots indicated that the LASSO regression models provided good fits to the data (Figure S5a–d), further details of which are shown in Table S3. LASSO regression selected BIO1, BIO5, soil pH value and NH_4_^+^‐N concentration as the four best predictors for the OTU richness of plant pathogenic fungal genera, with positive regression coefficients being recorded for each predictor except NH_4_^+^‐N concentration (Table 1). ANOVA indicated that BIO1 and pH value were significant predictors for the OTU richness of plant pathogenic fungi, with a highly significant effect of the former factor (Table 1). Univariate regression models showed highly significant positive linear associations between the OTU richness of plant pathogens and both BIO1 and BIO5, each with coefficients of 0.72–0.74, a significant positive linear association with soil pH value, and a quadratic association with NH_4_^+^‐N concentration (Figure 2a–d). The OTU and ASV richness of phytopathogenic fungi were closely associated with each other (Spearman's rank correlation coefficient = 0.990, *p* < 0.001), and LASSO regression hence selected the same four predictors for the ASV richness of the guild as those selected for OTU richness, with a highly significant positive effect of BIO1 and a significant positive effect of pH value (Table S4). Univariate regressions between each of these four predictor variables and the ASV richness of plant pathogenic fungi were similar to those between the variables and OTU richness (*c.f*. Figures 2a–d and S6a–d). BIO1, BIO6, BIO12, and soil pH value were selected by LASSO regression as the four best predictors for the relative abundance of plant pathogens, with ANOVA showing BIO1 and BIO12 to be highly significant and marginally significant predictors, respectively (Table 1). Univariate regression models showed significant positive linear associations between the relative abundance of plant pathogens and BIO1, BIO6 and soil pH value, and a quadratic association with BIO12 (Figure 2e–h).

**TABLE 1:** Intercepts and coefficients for predictors of the operational taxonomic unit richness and relative abundances of plant and animal pathogenic fungi derived from LASSO regression.

	Plant pathogenic fungi		Animal pathogenic fungi	
	Richness (no. OTUs)	Relative abundance (%)	Richness (no. OTUs)	Relative abundance (%)
Intercept	2.102 × 10^0^	3.037 × 10^−1^	2.483 × 10^0^	2.477 × 10^−1^
BIO1	6.221 × 10^−1^ ***	5.670 × 10^−3^ ***	2.879 × 10^−1^ ***	0
BIO4	0	0	0	0
BIO5	2.577 × 10^−2^	0	8.237 × 10^−2^	1.285 × 10^−2^ **
BIO6	0	6.970 × 10^−3^	0	0
BIO7	0	0	0	0
BIO12	0	−1.169 × 10^−2^ ^a^	−7.994 × 10^−1^ **	−6.340 × 10^−2^ ***
BIO15	0	0	0	0
pH value	1.047 × 10^0^ *	1.197 × 10^−2^	7.704 × 10^−1^ *	0
C/N ratio	0	0	0	3.647 × 10^−3^ ^a^
NO_3_^−^‐N	0	0	0	0
NH_4_^+^‐N	−1.815 × 10^−2^	0	0	4.222 × 10^−3^ *
TDN	0	0	0	0
TDOC	0	0	0	0

*Note:* Data shown are intercepts and coefficients with *λ* set to 1.719 × 10^−1^, 2.521 × 10^−2^, 2.989 × 10^−1^, and 1.329 × 10^−2^ for the richness and relative abundance of plant pathogenic fungi and the richness and relative abundance of animal pathogenic fungi, respectively. Note that LASSO regression models for relative abundances were fitted on Hellinger‐transformed data. Asterisks denote the significance of predictors derived from analysis of variance.

Abbreviations: OTUs, operational taxonomic units; TDN, total dissolved nitrogen; TDOC, total dissolved organic carbon.

^a^ *p* < 0.06.

* *p* < 0.05.

** *p* < 0.01.

*** *p* < 0.001.

**FIGURE 3:**
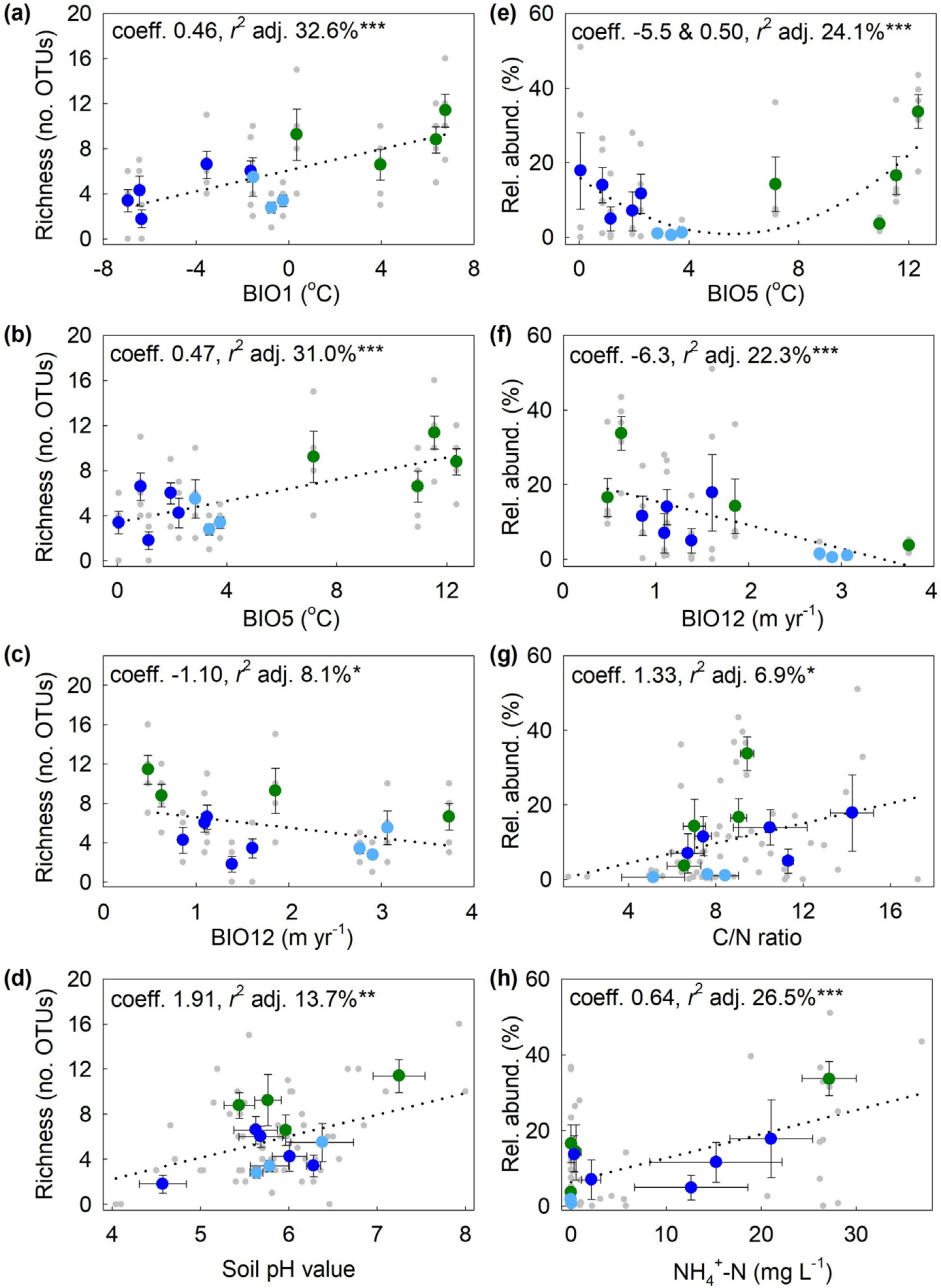
The OTU richness and relative abundance of soilborne animal pathogenic fungi as functions of climatic and edaphic factors selected by LASSO regression. (a–d) OTU richness as functions of BIO1, BIO5, BIO12, and soil pH value. (e–h) Relative abundance as functions of BIO5, BIO12, C/N ratio, and NH_4_^+^‐N concentration. The first and second coefficients shown in panel e are linear and squared components, respectively. Sampling sites are color‐coded for geographical region (see key in Figure 1b). Abbreviations and notation as in Figure 2.

LASSO regression selected BIO1, BIO5, BIO12, and pH value as the four best predictors for the OTU richness of animal pathogenic fungi, with positive coefficients for each predictor except BIO12 (Table 1). ANOVA showed BIO1, BIO12, and soil pH value to be significant predictors for the OTU richness of animal pathogens, with, again, a highly significant effect of BIO1 (Table 1). Univariate regression models similarly indicated highly significant positive linear associations between the OTU richness of animal pathogenic fungi and BIO1 and BIO5, but with lower coefficients than those recorded for phytopathogen richness (0.46–0.47; Figure 3a,b). Univariate models also indicated negative and positive linear associations with BIO12 and soil pH value, respectively (Figure 3c,d). As for phytopathogenic fungi, the OTU and ASV richness of animal pathogenic fungi were closely correlated with each other (Spearman's rank correlation coefficient = 0.960, *p* < 0.001), with LASSO regression selecting the same four predictor variables for the ASV richness of these fungi as those selected for OTU richness, but with a non‐significant (*p* > 0.05) effect of soil pH value (Table S4). Univariate regressions between each of these four predictor variables and the ASV richness of animal pathogenic fungi were similar to those between the variables and OTU richness (*c.f*. Figures 3a–d and S6e–h). LASSO regression selected BIO5, BIO12, C/N ratio, and NH_4_^+^‐N concentration as the four best predictors for the relative abundance of animal pathogens, with ANOVA indicating a highly significant effect of BIO12, significant effects of BIO5 and NH_4_^+^‐N concentration and a marginally significant influence of C/N ratio (Table 1). Univariate regression models showed a highly significant quadratic relationship between the relative abundance of animal pathogens and BIO5, a highly significant negative linear relationship with BIO12, and positive linear relationships with C/N ratio and NH_4_^+^‐N concentration (Figure 3e–h).

### 3.3 Predicted End‐Of‐Century Climate and Soilborne Fungal Pathogen OTU Richness and Abundance

**FIGURE 4:**
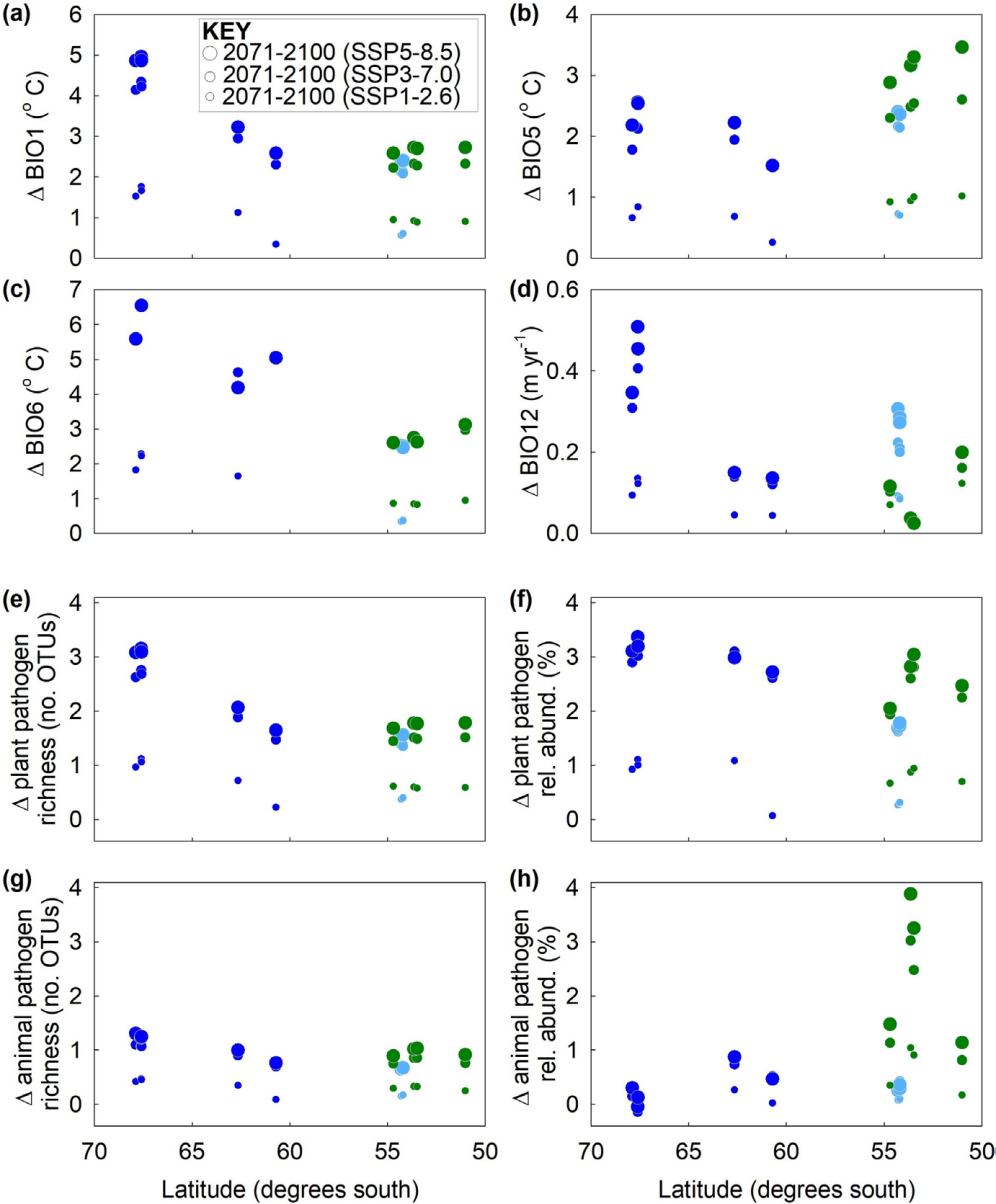
Projected changes to climatic factors and the OTU richness and relative abundance of soilborne pathogenic fungi along the latitudinal transect under three climate forcing scenarios. (a–d) Projected changes to BIO1, BIO5, BIO6, and BIO12 by 2071–2100 relative to 1981–2010 under the SSP1‐2.6, SSP3‐7.0, and SSP5‐8.5 climate forcing scenarios. (e–h) Changes to the OTU richness and relative abundance of plant and animal pathogenic fungi by 2071–2100 under the SSP1‐2.6, SSP3‐7.0, and SSP5‐8.5 climate forcing scenarios. Note that mean values are shown for each of the 12 sites and that all *x*‐ and *y*‐axes of panels e–h are identically scaled. Sizes of circles in a–h represent different scenarios (see key in panel a). Sampling sites are color‐coded for geographical region (see key in Figure 1b). Abbreviations as in Figure 1.

Climate projections under the SSP1‐2.6 scenario indicated increases in BIO1, BIO5, and BIO6 of 0.3°C–2.3°C along the transect by 2071–2100 (Figure 4a–c). In contrast, the SSP3‐7.0 and SSP5‐8.5 scenarios led to larger projected increases in BIO1, BIO5, and BIO6 of 1.5°C–6.6°C, with the largest rises in BIO1 and BIO6 at sites 1–3 in southern Maritime Antarctica (Figure 4a–c). For annual precipitation, although the SSP1‐2.6 scenario led to nominal projected increases in BIO12 of 0.04–0.14 m year^−1^ along the transect, the SSP3‐7.0 and SSP5‐8.5 scenarios caused BIO12 to increase by 0.10–0.51 m year^−1^ at ten sites, with the largest rises at sites 1–3, and negligible increases in this factor of 0.02–0.04 m year^−1^ at sites 10 and 11 in Patagonia (Figure 4d).

The projected rises in air temperatures and annual precipitation under the SSP1‐2.6 scenario caused nominal predicted changes to the OTU richness and relative abundance of plant pathogenic genera, with end‐of‐century increases in these parameters of 0.2–1.1 OTUs and 0.1%–1.1% along the transect, respectively (Figure 4e,f). In contrast, the SSP3‐7.0 and SSP5‐8.5 scenarios caused larger predicted changes to plant pathogen OTU richness. At sites 1–3, where soils are currently inhabited by 2.5–3.4 OTUs of phytopathogens, the rises in BIO1 and BIO5 under these two scenarios led to predicted increases of 2.6–3.2 OTUs of these fungi by 2071–2100 (Figure 4e), approximately doubling present‐day phytopathogen OTU richness in these soils. In soils at the other nine sites, which are currently inhabited by 4.8–14.0 OTUs of phytopathogenic fungi, the SSP3‐7.0 and SSP5‐8.5 scenarios caused smaller predicted changes to plant pathogen richness of 1.4–2.1 OTUs (Figure 4e). Similar impacts of the SSP3‐7.0 and SSP5‐8.5 scenarios were predicted on the relative abundances of plant pathogenic fungi. In the soils at sites 1–3, where phytopathogens on average currently account for 3.4% of the soil fungal community, the predicted changes in climate under the two scenarios caused increases of 2.9%–3.4% in the abundance of phytopathogens (Figure 4f), approximately doubling current phytopathogen abundance in these soils. At other sites where plant pathogens constitute 5.1%–35.0% of the community, the SSP3‐7.0 and SSP5‐8.5 scenarios led to projected increases in the relative abundance of phytopathogens of 1.6%–3.1% (Figure 4f).

The projected increases in BIO1, BIO5 and BIO12 under the SSP1‐2.6, SSP3‐7.0 and SSP5‐8.5 scenarios had comparatively weak effects on the OTU richness of animal pathogens, with 0.1–1.3 more OTUs of these fungi predicted in all soils along the transect by 2071–2100, and with negligible differences in the size of the effect between latitudes (Figure 4g). Compared with phytopathogen OTU richness, these smaller predicted increases in part reflected the smaller positive coefficient for BIO1, and the negative coefficient for BIO12, in the LASSO regression model for the OTU richness of the animal pathogen guild (Table 1). Similarly, projected changes under all three scenarios to BIO5 and BIO12, along with reductions in soil C/N ratio associated with increases in the former factor, led to a predicted 0.2% reduction in the relative abundance of animal pathogenic fungi at site 2 and to nominal 0.1%–1.5% increases in the abundance of the guild at sites 1, 3–9 and 12 (Figure 4h). Compared with plant pathogens, the negligible impacts of the three scenarios on the abundance of animal pathogenic fungi at these sites in part reflected the larger negative coefficient for BIO12 in the LASSO regression model for the guild (Table 1). However, at sites 10 and 11 in Patagonia, where animal pathogens currently account for 16.0%–33.6% of the soil fungal community, the SSP3‐7.0 and SSP5‐8.5 scenarios caused predicted end‐of‐century increases of 2.5%–3.9% in the relative abundance of animal pathogens (Figure 4h).

### 3.4 Soilborne Fungal Pathogens Indicative of Climatic and Edaphic Factors

**FIGURE 5:**
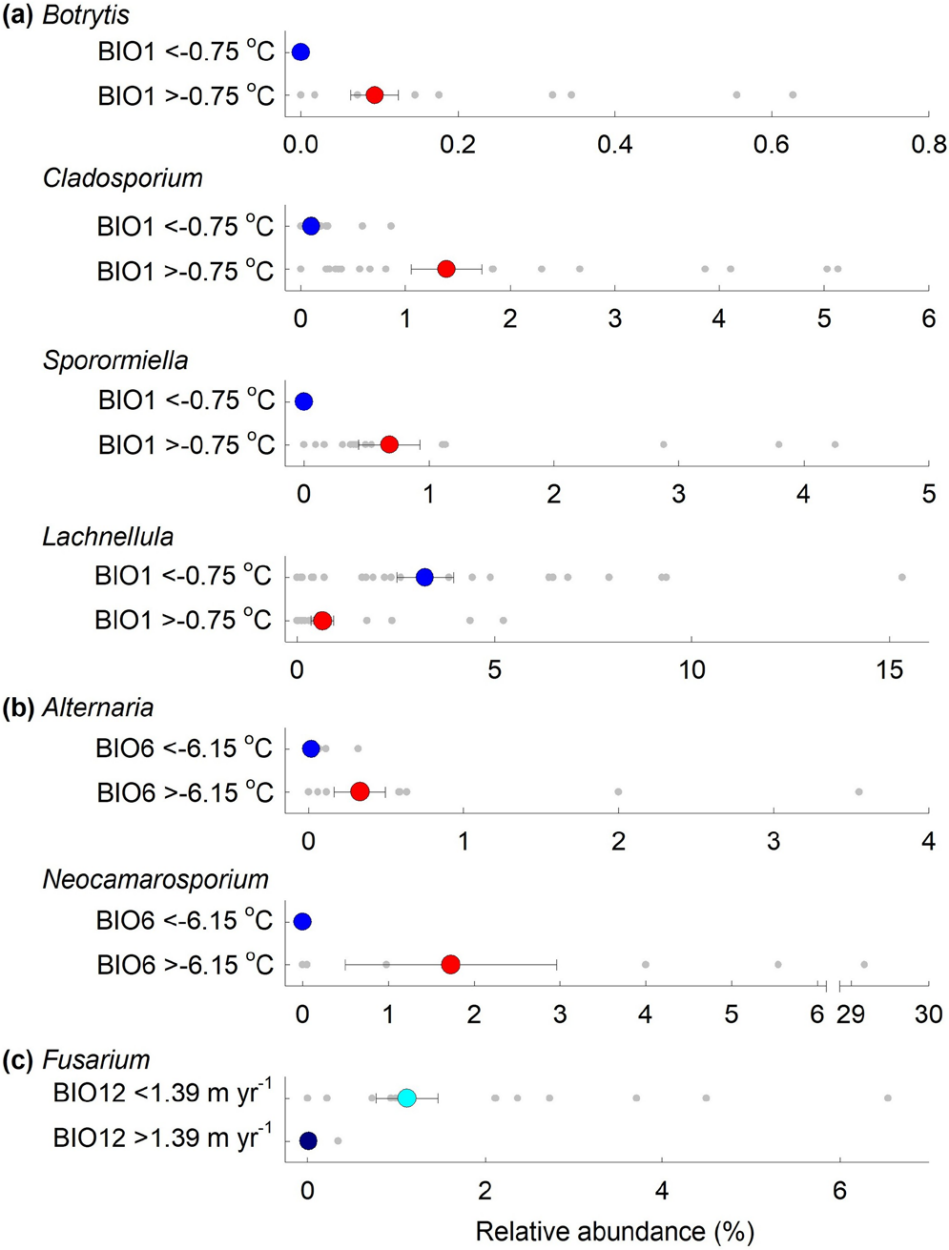
Soilborne plant pathogenic fungal genera indicative of climatic factors. (a) Mean relative abundances of *Botrytis*, *Cladosporium*, *Sporormiella*, and *Lachnellula* in soil at sites with BIO1 values of < −0.75°C and > −0.75°C, represented by blue and red circles, respectively. (b) Mean relative abundances of *Alternaria* and *Neocamarosporium* in soil at sites with BIO6 values of < −6.15°C and > −6.15°C, represented by blue and red circles, respectively. (c) Mean relative abundances of *Fusarium* in soil at sites with BIO12 values of < 1.39 m year^−1^ and > 1.39 m year^−1^, represented by cyan and dark blue circles, respectively. Values are means ± SEM and grey dots show individual data points. Each pairwise comparison was significantly different at *p* < 0.05 following Benjamini–Hochberg correction. Note that *Cladosporium*, *Alternaria*, and *Fusarium* also cause diseases of animals.

The OTUs were assigned to 105 genera that are broadly representative of fungal communities inhabiting barren Maritime Antarctic soils (Figure S7). Indicator analyses showed that, in broad agreement with their placements on a principal component loading plot (Figure S7b), the plant pathogenic genera *Botrytis*, *Cladosporium, Sporormiella*, and *Lachnellula* were each indicators for BIO1, with the former three genera, the second of which also causes diseases of animals, being indicative of soils from sites with BIO1 values of > −0.75°C (Figure 5a). *Botrytis* and *Sporormiella* were not recorded at sites 1–5 in Maritime Antarctica. Since BIO1, BIO5, and BIO6 were closely correlated with each other along the transect (Figure S1), *Botrytis*, *Cladosporium*, and *Sporormiella* were also significant indicators for sites with BIO5 values of > 3.35°C and BIO6 values of > −6.15°C (Figure S8a). In contrast, the genus *Lachnellula* was indicative of sites with BIO1 values of < −0.75°C (Figure 5a) and BIO6 values of < −6.15°C (Figure S8b). *Alternaria* and *Neocamarosporium*, the latter of which was absent from soils at sites 1–5, were both indicators for soils with BIO6 values of >− 6.15°C (Figure 5b). *Fusarium*, which also causes diseases of animals, was an indicator for mean annual precipitation, with the genus being more abundant in soils at sites with BIO12 values of < 1.39 m year^−1^ (Figure 5c), and notably at sites 10 and 11. Although phytopathogenic genera were less frequent indicators for edaphic factors, *Microdochium* and *Coleophoma* were marginally significant indicators for soils with C/N ratios of < 8.21 and NH_4_^+^‐N concentrations of < 0.07 mg L^−1^, respectively (Figure S8c,d).

**FIGURE 6:**
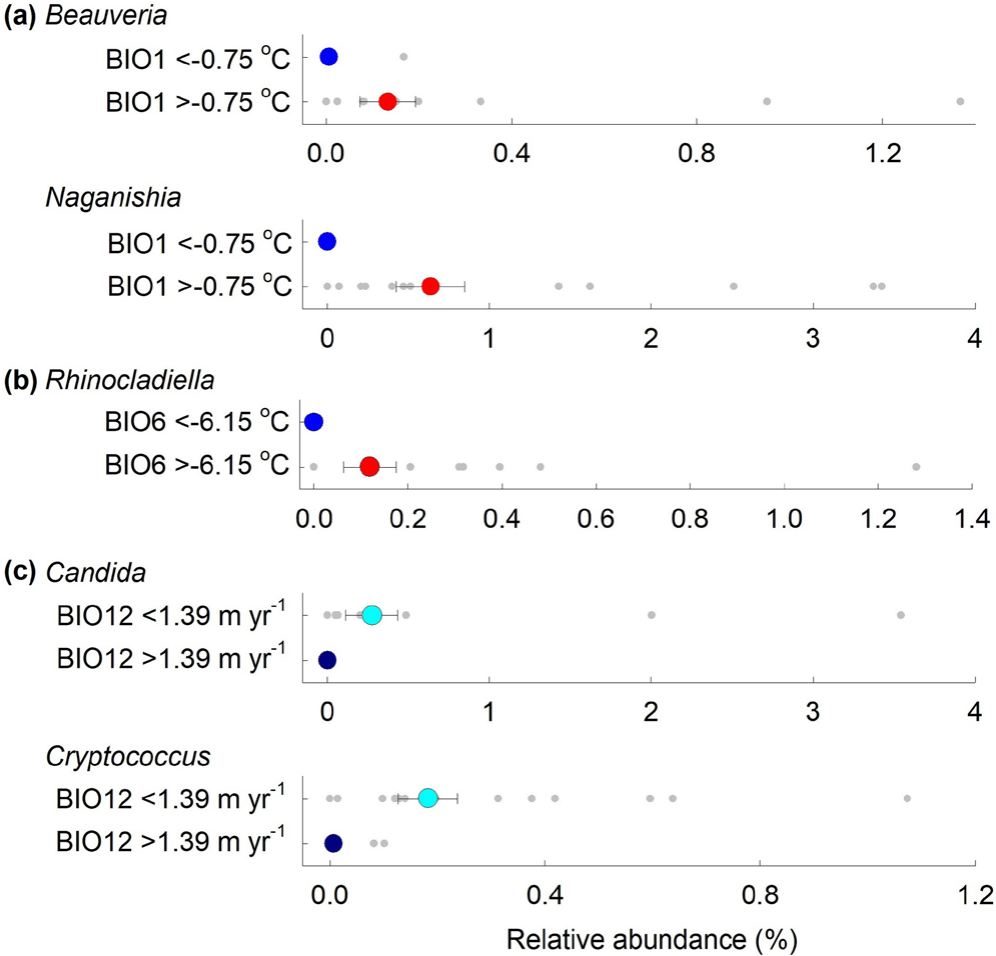
Soilborne animal pathogenic fungal genera indicative of climatic factors. (a) Mean relative abundances of *Beauveria* and *Naganishia* in soil at sites with BIO1 values of < −0.75°C and > −0.75°C, represented by blue and red circles, respectively. (b) Mean relative abundances of *Rhinocladiella* in soil at sites with BIO6 values of < −6.15°C and > −6.15°C, represented by blue and red circles, respectively. (c) Mean relative abundances of *Candida* and *Cryptococcus* in soil at sites with BIO12 values of < 1.39 m year^−1^ and > 1.39 m year^−1^, represented by cyan and dark blue circles, respectively. Values are means ± SEM and grey dots show individual data points. Each pairwise comparison was significantly different at *p* < 0.05 following Benjamini–Hochberg correction.

For the animal pathogenic genera, *Beauveria* and *Naganishia* were indicative of soils with BIO1 values of > −0.75°C (Figure 6a). Both genera were absent from soils at sites 1–5 in Maritime Antarctica and *Naganishia* was not recorded in soils at sites 7–9 in sub‐Antarctica. Owing to the close correlation between BIO1 and BIO5 (Figure S1), *Beauveria* and *Naganishia* were also representative of soils with BIO5 values of > 3.35°C (Figure S8e). *Rhinocladiella*, which was not recorded in soils at sites 1–5 and 7–9, was indicative of soils sampled from sites with BIO6 values of > −6.15°C (Figure 6b). The animal pathogenic yeasts *Candida* and *Cryptococcus* were both indicators for sites receiving < 1.39 m year^−1^ of precipitation (Figure 6c), with both genera being frequent in soil at site 10 in Patagonia. Although indicator analyses showed that other animal pathogenic genera, notably *Thelebolus*, but also *Penicillium* and *Pseudogymnoascus*, were not indicative of precipitation, the principal component score and loading plots showed that they were frequent in soils at sites 10 or 11 (Figure S7a,b). As for the phytopathogenic genera, animal pathogens were less frequent indicators for edaphic than climatic factors. However, *Pseudogymnoascus* was indicative of soils with total dissolved nitrogen concentrations of > 2.62 mg L^−1^, and *Cladophialophora* was a marginally significant indicator for soils with total dissolved organic carbon concentrations of < 25.07 mg L^−1^ (Figure S8f,g).

## 4 Discussion

Our findings show strong positive associations between air temperature and the richness or relative abundance of soilborne fungi assigned to genera causing, or associated with, diseases of plants or animals in barren Patagonian and Antarctic soils, most probably reflecting shifts towards fungal dispersal strategies, and in particular the release of spores to the atmosphere, at higher temperatures (Abrego et al. 2024; Newsham et al. 2016). These associations held true for both OTU and ASV richness, indicating similar effects of air temperature on approximate species‐level and genetic diversity, respectively. They suggest that end‐of‐century climate change projected under the SSP3‐7.0 and SSP5‐8.5 scenarios (IPCC 2021; Riahi et al. 2017) will force increases to the present‐day richness and abundance of soilborne fungal pathogens in Antarctica and Patagonia, and, by inference, the frequency of the diseases that these fungi cause. Notably, in southern Maritime Antarctic soils, which, owing principally to low air temperatures, are currently inhabited by relatively few pathogenic fungi, the analyses here forecast approximate doublings to the present‐day OTU richness and abundance of phytopathogens by 2071–2100, with diminishing effects on the guild at lower latitudes.

As in similar studies, the climate data used as predictors here were derived from global databases, with the resolution of these data affecting the outcomes of the analyses. Nevertheless, our findings align broadly with those from other studies predicting climate change to influence the taxonomic diversity and frequency of soilborne plant pathogenic fungi on continents other than Antarctica (Delgado‐Baquerizo et al. 2020; Mikryukov et al. 2023; Tedersoo et al. 2014; Větrovský et al. 2019; but see Luo et al. 2025). A previous study that included soils from Antarctica in a global phytopathogen assessment—which similarly identified climate as an important driver of fungal distribution—predicted increases under future climate change scenarios in the relative abundances of seven out of the 10 most abundant fungal genera that it recorded in soils (Li et al. 2023). However, most probably reflecting the strong influence of dense vegetation cover on the taxonomic composition of soil fungal communities (Newsham et al. 2021), there is little taxonomic overlap between this and the present study, with eight of these 10 genera, including the crop pathogens *Erysiphe* and *Pseudocercospora*, being absent from the barren soils studied here. Hence, our study closes an important knowledge gap regarding which taxa of phytopathogenic fungi are likely to become more prevalent in the barren soils of Antarctica as the climate of the region changes and plant populations, including those of *Colobanthus quitensis*, expand into these soils (Cannone et al. 2022; Grobe et al. 1997).

As in similar previous studies (e.g., Delgado‐Baquerizo et al. 2020; Li et al. 2023), our analyses were made at the genus level, and hence not all of the OTUs recorded here will necessarily cause disease. Nevertheless, assuming that increased taxonomic richness and relative abundance of soilborne fungi assigned to pathogenic genera will lead to higher frequencies of host infections, and, in the light of the efficient airborne dispersal of fungal propagules (Abrego et al. 2024; Bebber et al. 2013), then which plant diseases can be expected to alter in frequency in warmer and less arid Patagonian and Antarctic soils? Broadly, our observations indicate that *Fusarium*, a genus consisting of aggressive phytopathogens causing vascular wilts, head blight and root and stem rots (Armer et al. 2024), will decline in frequency as mean annual precipitation increases in Antarctica and Patagonia. Similarly, they suggest that *Lachnellula*, which causes cankers (Yde‐Andersen 1980), will also become less abundant in soils as air temperatures rise along the transect studied here. However, the majority of our findings point to rising air temperatures forcing increases, rather than decreases, to the frequencies of soilborne phytopathogenic fungi in Maritime Antarctica. They indicate increased frequencies in soils of the aggressively necrotrophic genus *Botrytis*, which causes grey molds (Dean et al. 2012; Williamson et al. 2007), and of *Neocamarosporium* and *Sporormiella*, which cause stem necroses and leaf spots (Do Amaral et al. 2005; Yin et al. 2024), as air temperatures rise in the region. Additionally, they show that increased frequencies of leaf spots, scabs and blights caused by *Cladosporium* and the aggressive phytopathogen *Alternaria* (Delgado‐Baquerizo et al. 2020; Kodama 2019; Thomma et al. 2005) can be anticipated as air temperatures rise across Maritime Antarctica.

Whilst approximate doublings to the present‐day richness of southern Maritime Antarctic soilborne phytopathogenic fungal communities consisting of 2.5–3.4 OTUs may not substantially increase their absolute taxonomic diversity, it should be borne in mind that single species of novel fungal pathogens, typically introduced to new environments through geographic jumps facilitated by human dispersal (Engering et al. 2013), have the capacity to infect naïve plant hosts and radically alter terrestrial ecosystems (Fones et al. 2017; Harvell et al. 2002). Notable examples from temperate environments of such pathogens include species of *Cryphonectria* and *Ophiostoma*, which almost eradicated *Castanea* and *Ulmus* species from North America and Europe (Agrios 2009; Harvell et al. 2002), and a species of the root‐infecting oomycete *Phytophthora*, which transformed native Australasian *Eucalyptus* forest to monocot‐dominated savanna (Weste and Marks 1987). The introduction of single species of phytopathogens to the soils of southern Maritime Antarctica, either through human activities or on air currents (Abrego et al. 2024; Convey and Peck 2019), could have similar disproportionately large impacts on the region's flora. Maritime Antarctic vegetation is already predisposed to fungal infections, caused by many months each year spent under snowpack at subzero temperatures and at photosynthetic photon flux densities close to zero during midwinter or under > 0.2 m of snow cover (Newsham 2010), which halt photosynthesis, exhaust reserves and weaken defences to pathogens (Tojo and Newsham 2012). Primary infections in Antarctic bryophytes are often caused by snow moulds, notably the oomycete *Globisporangium* (formerly assigned to *Pythium*), but also true fungi such as *Microdochium* and *Typhula* (Hoshino 2025), which are followed by secondary infections attributable to a range of ascomycetes, including *Cladosporium* (Rosa et al. 2020). Together with physiological stress caused by summertime heatwaves (Desaint et al. 2021; Robinson et al. 2020), increased frequencies of infections caused by these and other fungi are likely to diminish bryophyte and vascular plant fitness in warmer Maritime Antarctic soils (Bridge et al. 2008; Hoshino 2025).

Compared with phytopathogens, our analyses forecast weaker effects of climate change on the richness and abundance of soilborne animal pathogenic genera, in part reflecting the smaller positive coefficients for air temperature and larger negative coefficients for precipitation in the LASSO regression models for these fungi. Nevertheless, the indicator analyses showed that rising air temperatures are likely to increase the frequency of *Beauveria* in soils, posing a potential threat to invertebrates, the most abundant and diverse terrestrial animals in Antarctica (Convey and Biersma 2024), of which the genus is an important pathogen (Boomsma et al. 2014). It is questionable whether increased abundance of soilborne pathogenic fungi might pose a similar risk to vertebrates, which, in principle, should be protected from infection by the inability of most fungi to grow at temperatures exceeding ~37°C (Robert and Casadevall 2009). Nevertheless, fungal infections in Antarctic seals and penguins are not infrequent, with *Alternaria*, *Cladosporium*, and *Naganishia* colonising the nasal, oral, and cloacal mucosa and feathers of these animals (Brito Devoto et al. 2022). Although the patchy distribution of marine vertebrate colonies in Antarctica is likely to slow the transmission of these pathogens through host limitation—with, for example, fewer than 270 colonies of Adélie penguins present across the entire continent (Che‐Castaldo et al. 2017)—the increased occurrence in a warmer Maritime Antarctic of cutaneous, subcutaneous, and cerebral phaeohyphomycosis and cryptococcosis caused by pathogenic members of these genera (Li and de Hoog 2009; Oliveira et al. 2021), and of *Rhinocladiella* (Revankar and Sutton 2010), cannot be discounted. In humans, and particularly in immunosuppressed individuals, soilborne fungi in the region may hence pose a risk to health (de Sousa et al. 2017).

The negative coefficients for precipitation in the LASSO regression models for the richness of animal pathogenic fungi and the relative abundances of both pathogenic guilds run counter to the normally positive effects of precipitation on the taxonomic diversity and frequency of soilborne fungi (e.g., Mikryukov et al. 2023; Tedersoo et al. 2014). These observations possibly reflect the xerotolerance of microbes in arid regions. Several animal pathogenic genera, notably *Thelebolus*, but also *Penicillium*, *Pseudogymnoascus, Fusarium*, *Candida*, and *Cryptococcus*, were found to be frequent in soils at sites 10 or 11, which receive 0.48–0.63 m year^−1^ of precipitation. A previous study has shown Antarctic isolates of *Thelebolus*, *Penicillium*, and *Pseudogymnoascus* to grow at a water activity (*a*_*w*_) of 0.66 (Coelho et al. 2021), at below the limit of water availability considered to be hostile for life (≤ 0.72 *a*_*w*_; Williams and Hallsworth 2009) and at close to the absolute lower limit for extreme xerophilic fungal growth (~0.63 *a*_*w*_; Stevenson et al. 2015). Furthermore, *Fusarium*, *Candida* and *Cryptococcus*, species of which are listed as high priority or critical human pathogens (Rodrigues and Nosanchuk 2023; World Health Organization 2022), have been shown to be abundant in arid soils at high latitudes (di Menna 1966; Newsham et al. 2021). Increased frequencies of xerotolerant or xerophilic members of these genera in the arid soils studied here may hence account for the negative coefficients recorded for precipitation in the LASSO regression models, and particularly the model for the relative abundance of animal pathogens. Despite the negative coefficient for precipitation in this model, the SSP3‐7.0 and SSP5‐8.5 climate forcing scenarios nevertheless caused projected 2.5%–3.9% increases to the relative abundances of animal pathogens at sites 10 and 11. These sites, where the guild currently accounts for up to a third of the soil fungal community, are hence apparently at moderate future risk from diseases caused by animal pathogenic fungi.

In broad agreement with previous regional and global studies of soil fungi (Li et al. 2023; Newsham et al. 2016; Tedersoo et al. 2014; Větrovský et al. 2019), we found edaphic factors to explain lower amounts of variance in soil pathogenic fungal diversity and relative abundance than climatic factors, with ANOVA usually indicating less statistically significant fits for edaphic than for climatic factors, and fungal genera being only marginally significant indicators for C/N ratio and the concentrations of NH_4_^+^‐N and TDOC in soil. The former edaphic factor, which was positively associated with the relative abundance of animal pathogenic fungi, was adjusted in our predictive analyses to account for future increases in maximum summertime air temperature, reflecting the anticipated reductions in this ratio as warming accelerates organic matter decomposition in Maritime Antarctic soils (Bokhorst et al. 2007; Newsham et al. 2016). Soil pH value was positively associated with the richness of both plant and animal pathogenic fungi, confirming the importance of soil reaction for fungal diversity worldwide (Delgado‐Baquerizo et al. 2020; Newsham et al. 2021; Tedersoo et al. 2014; Větrovský et al. 2019). Whilst we cannot discount the possibility that increasing air temperature and precipitation in Maritime Antarctica and Patagonia might affect soil pH value through mechanisms not captured in our dataset, studies across transects through these regions over which mean annual air temperature alters by 7°C–8°C show no changes to soil pH value, with field experiments also showing no change to the pH value of barren Antarctic soil warmed by 1°C with open top chambers for 5 years (Horrocks et al. 2020; Newsham et al. 2016, 2022). Consequently, soil pH value was included, but was held constant, in our predictive analyses.

Soil NH_4_^+^‐N concentration was similarly held constant in the predictive analyses. Direct effects of increasing air temperatures on this edaphic factor along the transect studied here are unlikely, since, although permafrost thaw might elevate soil NH_4_^+^‐N concentrations (Hansen and Elberling 2023; Keuper et al. 2012), total nitrogen concentrations in barren soils at high latitudes are so low, both in the active layer and in underlying permafrost (e.g., 0.02%–0.07%; Newsham et al. 2022; Otero et al. 2013; Roberts et al. 2009), that negligible increases in NH_4_^+^‐N concentrations from melting permafrost are unlikely to influence the richness and relative abundance of soilborne fungal pathogens. It remains possible that future changes to the sizes of seal and seabird colonies, which emit windborne nitrogen and are the main source of inorganic forms of the element in nearby Maritime Antarctic soils (Bokhorst et al. 2019), might indirectly affect the richness of phytopathogenic fungi and the relative abundance of animal pathogenic fungi through their effects on soil NH_4_^+^‐N concentration. However, without reliable estimates of changes to populations of marine vertebrates, and the precise relationship between the numbers of these animals and inorganic nitrogen concentration in soils downwind of colonies, accurately predicting changes to soil NH_4_^+^‐N concentration, and subsequent impacts on the diversity and abundance of soilborne fungal pathogens, is currently not feasible.

## 5 Conclusions

Assuming the subordination of environmental concerns by the governments of nations contributing significantly to global greenhouse gas emissions (Garcia‐Soto 2025), and low priorities amongst other nations for addressing these concerns (Riahi et al. 2017), it is not unlikely that mean annual air temperature over Chile and Antarctica will rise by 4°C–5°C during the next seven decades (Bracegirdle et al. 2020; Williams 2017). Our findings indicate that rises in air temperature of this magnitude will force approximate doublings to the OTU richness and relative abundance of soilborne phytopathogenic fungi at high latitudes in Maritime Antarctica, giving rise to soils with pathogenic fungal burdens similar to those in present‐day sub‐Antarctica and southern Patagonia. Additionally, they point to increased abundances in Maritime Antarctic soils of phytopathogenic fungal genera characteristic of warmer habitats closer to the equator, including those causing grey molds, blights, scabs and leaf spots. The increased incidence of these, and potentially other, fungal diseases poses a threat to Maritime Antarctic plants as they colonize barren soils, chiefly through reductions in primary productivity but also through shifts in community composition and genetic diversity (Fisher et al. 2012; Harvell et al. 2002; Singh et al. 2023). As on other continents (Agrios 2009; Bebber et al. 2013, 2014; Delgado‐Baquerizo et al. 2020; Weste and Marks 1987), such changes may have far‐reaching consequences for the terrestrial ecosystems of the region by the end of the 21st century.

## Author Contributions

**Marco A. Molina‐Montenegro:** writing – review and editing, funding acquisition, supervision. **Gabriel I. Ballesteros:** writing – review and editing. **Ian S. Acuña‐Rodríguez:** writing – review and editing, investigation. **Elisabeth M. Biersma:** investigation, writing – review and editing. **Kevin K. Newsham:** conceptualization, writing – original draft, writing – review and editing, funding acquisition, supervision, formal analysis, data curation, investigation, visualization. **Reti Ranniku:** investigation, writing – review and editing. **Cristian Torres‐Díaz:** writing – review and editing, investigation, funding acquisition. **Anders Priemé:** funding acquisition, writing – review and editing, investigation. **William P. Goodall‐Copestake:** conceptualization, investigation, funding acquisition, writing – review and editing, methodology, supervision, data curation, formal analysis. **Gilda Varliero:** methodology, investigation, writing – review and editing, formal analysis. **Izzy Newsham:** methodology, software, writing – review and editing. **Peter Convey:** writing – review and editing, supervision, funding acquisition.

## Funding

This work was funded by a joint NERC‐CONICYT award (grant refs. NE/P003079/1 and PII20150126) and by an award from the Danish National Research Foundation (VOLT, DNRF 168).

## Conflicts of Interest

The authors declare no conflicts of interest.

## Supporting information


**Figure S1:** Correlogram showing Spearman's correlation coefficients for associations between latitude and the climatic and edaphic factors used as predictor variables.
**Figure S2:** Soil C/N ratio as a function of maximum air temperature of the warmest month (BIO5) across the latitudinal transect.
**Figure S3:** The pH value of barren Maritime Antarctic soil treated with open top chambers for 5 years.
**Figure S4:** Changes to climatic and edaphic factors across the latitudinal transect.
**Figure S5:** Summary of data distributions.
**Figure S6:** The amplicon sequence variant richness of soilborne plant and animal pathogenic fungi as functions of climatic and edaphic factors selected by LASSO regression.
**Figure S7:** Principal component analysis of associations between climatic and edaphic factors and the abundances of pathogenic fungal genera.
**Figure S8:** Soilborne plant and animal pathogenic fungal genera indicative of climatic and edaphic factors.
**Table S1:** Genera assigned to plant or animal pathogen guilds.
**Table S2:** Climatic and edaphic factors used as predictors and their median values along the transect.
**Table S3:** Summary of the DNA sequencing data on which the analyses were based.
**Table S4:** Intercepts and coefficients for predictors of the amplicon sequence variant richness of plant and animal pathogenic fungi derived from LASSO regression.
**Appendix S1:** R script used to retrieve and analyse data.

## Data Availability

All data and code that are needed to evaluate the findings here are reported in the article, in the Supporting Information, or by Newsham et al. (2025). The Illumina DNA raw sequence data are uploaded to the Sequence Read Archive at the NCBI under Bioproject PRJNA1366494.

## References

[gcb70885-bib-0001] Abarenkov, K. , A. Zirk , T. Piirmann , et al. 2023. UNITE General FASTA Release for Fungi. UNITE Community. 10.15156/BIO/2938067.

[gcb70885-bib-0002] Abrego, N. , B. Furneaux , B. Hardwick , et al. 2024. “Airborne DNA Reveals Predictable Spatial and Seasonal Dynamics of Fungi.” Nature 631, no. 8022: 835–842. 10.1038/s41586-024-07658-9. 38987593 PMC11269176

[gcb70885-bib-0003] Agrios, G. N. 2009. “Plant Pathogens and Disease: General Introduction.” In Encyclopedia of Microbiology (Third Edition), edited by M. Schaechter , 613–646. Academic Press. 10.1016/B978-012373944-5.00344-8.

[gcb70885-bib-0004] Armer, V. J. , E. Kroll , M. Darino , D. Smith , M. Urban , and K. E. Hammond‐Kosack . 2024. “Navigating the *Fusarium* Species Complex: Host‐Range Plasticity and Genome Variations.” Fungal Biology 128, no. 8: 2439–2459. 10.1016/j.funbio.2024.07.004. 39653491

[gcb70885-bib-0005] Bebber, D. , M. Ramotowski , and S. J. Gurr . 2013. “Crop Pests and Pathogens Move Polewards in a Warming World.” Nature Climate Change 3, no. 11: 985–988. 10.1038/nclimate1990.

[gcb70885-bib-0006] Bebber, D. P. , T. Holmes , and S. J. Gurr . 2014. “The Global Spread of Crop Pests and Pathogens.” Global Ecology and Biogeography 23, no. 12: 1398–1407. 10.1111/geb.12214.

[gcb70885-bib-0007] Benjamini, Y. , and Y. Hochberg . 1995. “Controlling the False Discovery Rate: A Practical and Powerful Approach to Multiple Testing.” Journal of the Royal Statistical Society. Series B, Statistical Methodology 57, no. 1: 289–300. 10.1111/j.2517-6161.1995.tb02031.x.

[gcb70885-bib-0008] Bokhorst, S. , P. Convey , and R. Aerts . 2019. “Nitrogen Inputs by Marine Vertebrates Drive Abundance and Richness in Antarctic Terrestrial Ecosystems.” Current Biology 29, no. 10: 1721–1727. 10.1016/j.cub.2019.04.038. 31080085

[gcb70885-bib-0009] Bokhorst, S. , A. Huiskes , P. Convey , and R. Aerts . 2007. “Climate Change Effects on Organic Matter Decomposition Rates in Ecosystems From the Maritime Antarctic and Falkland Islands.” Global Change Biology 13, no. 12: 2642–2653. 10.1111/j.1365-2486.2007.01468.x.

[gcb70885-bib-0010] Boomsma, J. J. , A. B. Jensen , N. V. Meyling , and J. Eilenberg . 2014. “Evolutionary Interaction Networks of Insect Pathogenic Fungi.” Annual Review of Entomology 59: 467–485. 10.1146/annurev-ento-011613-162054. 24160418

[gcb70885-bib-0011] Bracegirdle, T. J. , G. Krinner , M. Tonelli , et al. 2020. “Twenty First Century Changes in Antarctic and Southern Ocean Surface Climate in CMIP6.” Atmospheric Science Letters 21, no. 9: e984. 10.1002/asl.984.

[gcb70885-bib-0012] Bridge, P. D. , K. K. Newsham , and G. J. Denton . 2008. “Snow Mould Caused by a *Pythium* sp.: A Potential Vascular Plant Pathogen in the Maritime Antarctic.” Plant Pathology 57, no. 6: 1066–1072. 10.1111/j.1365-3059.2008.01868.x.

[gcb70885-bib-0013] Brito Devoto, T. , M. Toscanini , K. Hermida Alava , et al. 2022. “Exploring Fungal Diversity in Antarctic Wildlife: Isolation and Molecular Identification of Culturable Fungi From Penguins and Pinnipeds.” New Zealand Veterinary Journal 70, no. 5: 263–272. 10.1080/00480169.2022.2087784. 35673970

[gcb70885-bib-0014] Brun, P. , N. E. Zimmermann , C. Hari , L. Pellissier , and D. N. Karger . 2022. “Global Climate‐Related Predictors at Kilometer Resolution for the Past and Future.” Earth System Science Data 14, no. 12: 5573–5603. 10.5194/essd-14-5573-2022.

[gcb70885-bib-0015] Callahan, B. J. , P. J. McMurdie , M. J. Rosen , A. W. Han , A. J. A. Johnson , and S. P. Holmes . 2016. “DADA2: High‐Resolution Sample Inference From Illumina Amplicon Data.” Nature Methods 13, no. 7: 581–583. 10.1038/nmeth.3869. 27214047 PMC4927377

[gcb70885-bib-0016] Camacho, C. , G. Coulouris , V. Avagyan , et al. 2009. “BLAST+: Architecture and Applications.” BMC Bioinformatics 10: 421. 10.1186/1471-2105-10-421. 20003500 PMC2803857

[gcb70885-bib-0017] Cannone, N. , F. Malfasi , S. E. Favero‐Longo , P. Convey , and M. Guglielmin . 2022. “Acceleration of Climate Warming and Plant Dynamics in Antarctica.” Current Biology 32, no. 7: 1599–1606. 10.1016/j.cub.2022.01.074. 35167803

[gcb70885-bib-0018] Casadevall, A. , and L.‐A. Pirofski . 2001. “Host‐Pathogen Interactions: The Attributes of Virulence.” Journal of Infectious Diseases 184, no. 3: 337–344. 10.1086/322044. 11443560

[gcb70885-bib-0019] Chao, A. , N. J. Gotelli , T. C. Hsieh , et al. 2014. “Rarefaction and Extrapolation With Hill Numbers: A Framework for Sampling and Estimation in Species Diversity Studies.” Ecological Monographs 84, no. 1: 45–67. 10.1890/13-0133.1.

[gcb70885-bib-0020] Chao, A. , K. H. Ma , and T. C. Hsieh . 2016. “iNEXT Online: Software for Interpolation and Extrapolation of Species Diversity. Program and User's Guide.” http://chao.stat.nthu.edu.tw/wordpress/software_download/inext‐online/.

[gcb70885-bib-0021] Che‐Castaldo, C. , S. Jenouvrier , C. Youngflesh , et al. 2017. “Pan‐Antarctic Analysis Aggregating Spatial Estimates of Adélie Penguin Abundance Reveals Robust Dynamics Despite Stochastic Noise.” Nature Communications 8: 832. 10.1038/s41467-017-00890-0. PMC5635117 29018199

[gcb70885-bib-0022] Coelho, L. d. C. , C. R. de Carvalho , C. A. Rosa , and L. H. Rosa . 2021. “Diversity, Distribution, and Xerophilic Tolerance of Cultivable Fungi Associated With the Antarctic Angiosperms.” Polar Biology 44, no. 2: 379–388. 10.1007/s00300-021-02799-3.

[gcb70885-bib-0023] Convey, P. , and E. M. Biersma . 2024. “Antarctic Ecosystems.” In Encyclopedia of Biodiversity (Third Edition), edited by S. M. Scheiner , 133–148. Academic Press. 10.1016/B978-0-12-822562-2.00058-X.

[gcb70885-bib-0024] Convey, P. , and L. S. Peck . 2019. “Antarctic Environmental Change and Biological Responses.” Science Advances 5, no. 11: eaaz0888. 10.1126/sciadv.aaz0888. 31807713 PMC6881164

[gcb70885-bib-0025] Davis, N. M. , D. Proctor , S. P. Holmes , D. A. Relman , and B. J. Callahan . 2017. “Simple Statistical Identification and Removal of Contaminant Sequences in Marker‐Gene and Metagenomics Data.” bioRxiv: 221499. 10.1101/221499. PMC6298009 30558668

[gcb70885-bib-0026] De Cáceres, M. , and P. Legendre . 2009. “Associations Between Species and Groups of Sites: Indices and Statistical Inference.” Ecology 90, no. 12: 3566–3574. 10.1890/08-1823.1. 20120823

[gcb70885-bib-0027] de Sousa, J. R. P. , V. N. Gonçalves , R. A. de Holanda , et al. 2017. “Pathogenic Potential of Environmental Resident Fungi From Ornithogenic Soils of Antarctica.” Fungal Biology 121, no. 12: 991–1000. 10.1016/j.funbio.2017.09.005. 29122179

[gcb70885-bib-0028] Dean, R. , J. A. L. Van Kan , Z. A. Pretorius , et al. 2012. “The Top 10 Fungal Pathogens in Molecular Plant Pathology.” Plant Pathology 13, no. 4: 414–430. 10.1111/j.1364-3703.2011.00783.x. PMC6638784 22471698

[gcb70885-bib-0029] Delgado‐Baquerizo, M. , C. A. Guerra , C. Cano‐Díaz , et al. 2020. “The Proportion of Soil‐Borne Pathogens Increases With Warming at the Global Scale.” Nature Climate Change 10, no. 6: 550–554. 10.1038/s41558-020-0759-3.

[gcb70885-bib-0030] Desaint, H. , N. Aoun , L. Deslandes , F. Vailleau , F. Roux , and R. Berthomé . 2021. “Fight Hard or Die Trying: When Plants Face Pathogens Under Heat Stress.” New Phytologist 229, no. 2: 712–734. 10.1111/nph.16965. 32981118

[gcb70885-bib-0031] di Menna, M. E. 1966. “Yeasts in Antarctic Soils.” Antonie Van Leeuwenhoek 32: 29–38. 10.1007/BF02097443. 5296606

[gcb70885-bib-0032] Do Amaral, A. L. , F. K. Dal Soglio , M. L. De Carli , and J. F. Barbosa Neto . 2005. “Pathogenic Fungi Causing Symptoms Similar to *Phaeosphaeria* Leaf Spot of Maize in Brazil.” Plant Disease 89, no. 1: 44–49. 10.1094/PD-89-0044. 30795283

[gcb70885-bib-0033] Engering, A. , L. Hogerwerf , and J. Slingenbergh . 2013. “Pathogen‐Host‐Environment Interplay and Disease Emergence.” Emerging Microbes & Infections 2, no. 1: 1–7. 10.1038/emi.2013.5. PMC3630490 26038452

[gcb70885-bib-0034] Fisher, M. , D. Henk , C. Briggs , et al. 2012. “Emerging Fungal Threats to Animal, Plant and Ecosystem Health.” Nature 484, no. 7393: 186–194. 10.1038/nature10947. 22498624 PMC3821985

[gcb70885-bib-0035] Fones, H. N. , M. C. Fisher , and S. J. Gurr . 2017. “Emerging Fungal Threats to Plants and Animals Challenge Agriculture and Ecosystem Resilience.” Microbiology Spectrum 5, no. 2: 1–23. 10.1128/microbiolspec.funk-0027-2016. PMC11687465 28361733

[gcb70885-bib-0036] Friedman, J. , T. Hastie , and R. Tibshirani . 2010. “Regularization Paths for Generalized Linear Models via Coordinate Descent.” Journal of Statistical Software 33, no. 1: 1–22. 10.18637/jss.v033.i01. 20808728 PMC2929880

[gcb70885-bib-0037] Garcia‐Solache, M. A. , and A. Casadevall . 2010. “Global Warming Will Bring New Fungal Diseases for Mammals.” MBio 1, no. 1: 61. 10.1128/mbio.00061-10. PMC2912667 20689745

[gcb70885-bib-0038] Garcia‐Soto, C. 2025. “Reversing Climate Progress: Consequences and Solutions in the Wake of US Policy Rollbacks.” NPJ Climate Action 4, no. 1: 63. 10.1038/s44168-025-00247-0.

[gcb70885-bib-0039] Grobe, C. W. , C. T. Ruhland , and T. A. Day . 1997. “A New Population of *Colobanthus quitensis* Near Arthur Harbor, Antarctica: Correlating Recruitment With Warmer Summer Temperatures.” Arctic and Alpine Research 29, no. 2: 217–221. 10.1080/00040851.1997.12003235.

[gcb70885-bib-0040] Gusa, A. , V. Yadav , C. Roth , et al. 2023. “Genome‐Wide Analysis of Heat Stress‐Stimulated Transposon Mobility in the Human Fungal Pathogen *Cryptococcus deneoformans* .” Proceedings of the National Academy of Sciences of the United States of America 120, no. 4: e2209831120. 10.1073/pnas.2209831120. 36669112 PMC9942834

[gcb70885-bib-0041] Hansen, H. F. E. , and B. Elberling . 2023. “Spatial Distribution of Bioavailable Inorganic Nitrogen From Thawing Permafrost.” Global Biogeochemical Cycles 37: e2022GB007589. 10.1029/2022GB007589.

[gcb70885-bib-0042] Harvell, C. D. , C. E. Mitchell , J. R. Ward , et al. 2002. “Climate Warming and Disease Risks for Terrestrial and Marine Biota.” Science 296, no. 5576: 2158–2162. 10.1126/science.1063699. 12077394

[gcb70885-bib-0043] Hijmans, R. J. , R. Bivand , E. Cordano , K. Dyba , E. Pebesma , and M. D. Sumner . 2022. “Terra: Spatial Data Analysis. Version 1.7‐83.”

[gcb70885-bib-0044] Horrocks, C. A. , K. K. Newsham , F. Cox , M. H. Garnett , C. H. Robinson , and J. A. J. Dungait . 2020. “Predicting Climate Change Impacts on Maritime Antarctic Soils: A Space‐For‐Time Substitution Study.” Soil Biology and Biochemistry 141: 107682. 10.1016/j.soilbio.2019.107682.

[gcb70885-bib-0045] Hoshino, T. 2025. “Diversity, Geographical Distribution and Environmental Adaptations of Snow Molds.” Mycoscience 66, no. 6: 334–349. 10.47371/mycosci.2025.09.001. 41969827 PMC13062971

[gcb70885-bib-0046] Ihrmark, K. , I. T. M. Bödeker , K. Cruz‐Martinez , et al. 2012. “New Primers to Amplify the Fungal ITS2 Region—Evaluation by 454‐Sequencing of Artificial and Natural Communities.” FEMS Microbiology Ecology 82, no. 3: 666–677. 10.1111/j.1574-6941.2012.01437.x. 22738186

[gcb70885-bib-0047] IPCC . 2021. “Summary for Policymakers.” In Climate Change 2021: The Physical Science Basis. Contribution of Working Group I to the Sixth Assessment Report of the Intergovernmental Panel on Climate Change, 3–32. Cambridge University Press. 10.1017/9781009157896.001.

[gcb70885-bib-0048] Karger, D. , O. Conrad , J. Böhner , et al. 2017. “Climatologies at High Resolution for the Earth's Land Surface Areas.” Scientific Data 4: 170122. 10.1038/sdata.2017.122. 28872642 PMC5584396

[gcb70885-bib-0049] Keuper, F. , P. M. van Bodegom , and E. Dorrepaal . 2012. “A Frozen Feast: Thawing Permafrost Increases Plant‐Available Nitrogen in Subarctic Peatlands.” Global Change Biology 18, no. 6: 1998–2007. 10.1111/j.1365-2486.2012.02663.x.

[gcb70885-bib-0050] Kodama, M. 2019. “Evolution of Pathogenicity in *Alternaria* Plant Pathogens.” Journal of General Plant Pathology 85, no. 6: 471–474. 10.1007/s10327-019-00877-3.

[gcb70885-bib-0051] Li, D. M. , and G. S. de Hoog . 2009. “Cerebral Phaeohyphomycosis—A Cure at What Lengths?” Lancet Infectious Diseases 9, no. 6: 376–383. 10.1016/S1473-3099(09)70131-8. 19467477

[gcb70885-bib-0052] Li, P. , L. Tedersoo , T. W. Crowther , et al. 2023. “Global Diversity and Biogeography of Potential Phytopathogenic Fungi in a Changing World.” Nature Communications 14, no. 1: 6482. 10.1038/s41467-023-42142-4. PMC10576792 37838711

[gcb70885-bib-0053] Luo, W. , K. G. Peay , T. Gonçalves‐Souza , P. B. Reich , D. R. Zak , and K. Zhu . 2025. “Climate and Land‐Use Changes Predicted to Jointly Drive Soil Fungal Diversity Losses in One‐Third of North American Coniferous Forests.” Global Change Biology 31, no. 11: e70598. 10.1111/gcb.70598. 41204848 PMC12595595

[gcb70885-bib-0054] Mahon, M. B. , A. Sack , O. A. Aleuy , et al. 2024. “A Meta‐Analysis on Global Change Drivers and the Risk of Infectious Disease.” Nature 629, no. 8013: 830–836. 10.1038/s41586-024-07380-6. 38720068

[gcb70885-bib-0055] Martin, M. 2011. “Cutadapt Removes Adapter Sequences From High‐Throughput Sequencing Reads.” EMBnet.Journal 17, no. 1: 10–12. 10.14806/ej.17.1.200.

[gcb70885-bib-0056] McNeish, D. M. 2015. “Using Lasso for Predictor Selection and to Assuage Overfitting: A Method Long Overlooked in Behavioral Sciences.” Multivariate Behavioral Research 50, no. 5: 471–484. 10.1080/00273171.2015.1036965. 26610247

[gcb70885-bib-0057] Mikryukov, V. , O. Dulya , A. Zizka , et al. 2023. “Connecting the Multiple Dimensions of Global Soil Fungal Diversity.” Science Advances 9, no. 48: eadj8016. 10.1126/sciadv.adj8016. 38019923 PMC10686567

[gcb70885-bib-0058] Newsham, K. K. 2010. “The Biology and Ecology of the Liverwort *Cephaloziella varians* in Antarctica.” Antarctic Science 22, no. 2: 131–143. 10.1017/S0954102009990630.

[gcb70885-bib-0059] Newsham, K. K. , E. M. Biersma , A. Prieme , M. A. Molina‐Montenegro , and W. P. Goodall‐Copestake . 2025. Richness and Abundance of Plant and Animal Pathogenic Fungi in Patagonian, Sub‐Antarctic and Maritime Antarctic Soil Samples Collected January–February 2018 (Version 1.0) [Data Set]. NERC EDS UK Polar Data Centre. 10.5285/d93d9ae6-4eff-4d9a-9997-d0ed06c3df1b.

[gcb70885-bib-0060] Newsham, K. K. , M. L. Davey , D. W. Hopkins , and P. G. Dennis . 2021. “Regional Diversity of Maritime Antarctic Soil Fungi and Predicted Responses of Guilds and Growth Forms to Climate Change.” Frontiers in Microbiology 11: 615659. 10.3389/fmicb.2020.615659. 33574801 PMC7870798

[gcb70885-bib-0061] Newsham, K. K. , D. W. Hopkins , L. Carvalhais , et al. 2016. “Relationship Between Soil Fungal Diversity and Temperature in the Maritime Antarctic.” Nature Climate Change 6, no. 2: 182–186. 10.1038/nclimate2806.

[gcb70885-bib-0062] Newsham, K. K. , M. Misiak , W. P. Goodall‐Copestake , et al. 2022. “Experimental Warming Increases Fungal Alpha Diversity in an Oligotrophic Maritime Antarctic Soil.” Frontiers in Microbiology 13: 1050372. 10.3389/fmicb.2022.1050372. 36439821 PMC9684652

[gcb70885-bib-0063] Nguyen, N. H. , Z. Song , S. T. Bates , et al. 2016. “FUNGuild: An Open Annotation Tool for Parsing Fungal Community Datasets by Ecological Guild.” Fungal Ecology 20: 241–248. 10.1016/j.funeco.2015.06.006.

[gcb70885-bib-0064] Oliveira, L. S. d. S. , L. M. Pinto , M. A. P. De Medeiros , et al. 2021. “Comparison of *Cryptococcus gattii*/*neoformans* Species Complex to Related Genera (*Papiliotrema* and *Naganishia*) Reveal Variances in Virulence Associated Factors and Antifungal Susceptibility.” Frontiers in Cellular and Infection Microbiology 11: 642658. 10.3389/fcimb.2021.642658. 34277464 PMC8281300

[gcb70885-bib-0065] Otero, X. L. , S. Fernández , M. A. D. P. Hernandez , E. C. Nizoli , and A. Quesada . 2013. “Plant Communities as a Key Factor in Biogeochemical Processes Involving Micronutrients (Fe, Mn, Co, and Cu) in Antarctic Soils (Byers Peninsula, Maritime Antarctica).” Geoderma 195: 145–154. 10.1016/j.geoderma.2012.11.018.

[gcb70885-bib-0067] R Core Team . 2022. R: A Language and Environment for Statistical Computing. R Foundation for Statistical Computing. https://www.R‐project.org/.

[gcb70885-bib-0068] Revankar, S. G. , and D. A. Sutton . 2010. “Melanized Fungi in Human Disease.” Clinical Microbiology Reviews 23, no. 4: 884–928. 10.1128/cmr.00019-10. 20930077 PMC2952981

[gcb70885-bib-0069] Riahi, K. , D. P. van Vuuren , E. Kriegler , et al. 2017. “The Shared Socioeconomic Pathways and Their Energy, Land Use, and Greenhouse Gas Emissions Implications: An Overview.” Global Environmental Change 42: 153–168. 10.1016/j.gloenvcha.2016.05.009.

[gcb70885-bib-0070] Robert, V. A. , and A. Casadevall . 2009. “Vertebrate Endothermy Restricts Most Fungi as Potential Pathogens.” Journal of Infectious Diseases 200, no. 10: 1623–1626. 10.1086/644642. 19827944

[gcb70885-bib-0071] Roberts, P. , K. K. Newsham , R. D. Bardgett , J. F. Farrar , and D. L. Jones . 2009. “Vegetation Cover Regulates the Quantity, Quality and Temporal Dynamics of Dissolved Organic Carbon and Nitrogen in Antarctic Soils.” Polar Biology 32, no. 7: 999–1008. 10.1007/s00300-009-0599-0.

[gcb70885-bib-0072] Robinson, S. A. , A. R. Klekociuk , D. H. King , M. P. Rojas , G. E. Zúñiga , and D. M. Bergstrom . 2020. “The 2019/2020 Summer of Antarctic Heatwaves.” Global Change Biology 26, no. 6: 3178–3180. 10.1111/gcb.15083. 32227664

[gcb70885-bib-0073] Rodrigues, M. L. , and J. D. Nosanchuk . 2023. “Recognition of Fungal Priority Pathogens: What Next?” PLoS Neglected Tropical Diseases 17, no. 3: e0011136. 10.1371/journal.pntd.0011136. 36893096 PMC9997940

[gcb70885-bib-0074] Rosa, L. H. , J. R. P. de Sousa , G. C. A. de Menezes , et al. 2020. “Opportunistic Fungi Found in Fairy Rings Are Present on Different Moss Species in the Antarctic Peninsula.” Polar Biology 43, no. 5: 587–596. 10.1007/s00300-020-02663-w.

[gcb70885-bib-0075] Singh, B. K. , M. Delgado‐Baquerizo , E. Egidi , et al. 2023. “Climate Change Impacts on Plant Pathogens, Food Security and Paths Forward.” Nature Reviews Microbiology 21, no. 10: 640–656. 10.1038/s41579-023-00900-7. 37131070 PMC10153038

[gcb70885-bib-0076] Stevenson, A. , J. A. Cray , J. P. Williams , et al. 2015. “Is There a Common Water‐Activity Limit for the Three Domains of Life?” ISME Journal 9, no. 6: 1333–1351. 10.1038/ismej.2014.219. 25500507 PMC4438321

[gcb70885-bib-0077] Tedersoo, L. , M. Bahram , S. Põlme , et al. 2014. “Global Diversity and Geography of Soil Fungi.” Science 346, no. 6213: 1256688. 10.1126/science.1256688. 25430773

[gcb70885-bib-0078] Tedersoo, L. , V. Mikryukov , S. Anslan , et al. 2021. “The Global Soil Mycobiome Consortium Dataset for Boosting Fungal Diversity Research.” Fungal Diversity 111, no. 1: 573–588. 10.1007/s13225-021-00493-7.

[gcb70885-bib-0079] Thomma, B. P. H. J. , H. P. Van Esse , P. W. Crous , and P. J. G. M. De Wit . 2005. “ *Cladosporium fulvum* (Syn. *Passalora fulva*), a Highly Specialized Plant Pathogen as a Model for Functional Studies on Plant Pathogenic Mycosphaerellaceae.” Molecular Plant Pathology 6, no. 4: 379–393. 10.1111/j.1364-3703.2005.00292.x. 20565665

[gcb70885-bib-0080] Tibshirani, R. 1996. “Regression Shrinkage and Selection via the Lasso.” Journal of the Royal Statistical Society. Series B, Statistical Methodology 58, no. 1: 267–288. 10.1111/j.2517-6161.1996.tb02080.x.

[gcb70885-bib-0081] Tojo, M. , and K. K. Newsham . 2012. “Snow Moulds in Polar Environments.” Fungal Ecology 5, no. 4: 395–402. 10.1016/j.funeco.2012.01.003.

[gcb70885-bib-0082] Turner, J. , T. A. Lachlan‐Cope , G. J. Marshall , E. M. Morris , R. Mulvaney , and W. Winter . 2002. “Spatial Variability of Antarctic Peninsula Net Surface Mass Balance.” Journal of Geophysical Research. Atmospheres 107, no. D13: 4172. 10.1029/2001JD000755.

[gcb70885-bib-0083] Větrovský, T. , P. Kohout , M. Kopecký , et al. 2019. “A Meta‐Analysis of Global Fungal Distribution Reveals Climate‐Driven Patterns.” Nature Communications 10: 5142. 10.1038/s41467-019-13164-8. PMC6853883 31723140

[gcb70885-bib-0084] Wang, Q. , G. M. Garrity , J. M. Tiedje , and J. R. Cole . 2007. “Naïve Bayesian Classifier for Rapid Assignment of rRNA Sequences Into the New Bacterial Taxonomy.” Applied and Environmental Microbiology 73, no. 16: 5261–5267. 10.1128/AEM.00062-07. 17586664 PMC1950982

[gcb70885-bib-0085] Weste, G. , and G. C. Marks . 1987. “The Biology of *Phytophthora cinnamomi* in Australasian Forests.” Annual Review of Phytopathology 25: 207–229. 10.1146/annurev.py.25.090187.001231.

[gcb70885-bib-0086] White, T. J. , T. D. Bruns , S. B. Lee , and J. W. Taylor . 1990. “Amplification and Direct Sequencing of Fungal Ribosomal RNA Genes for Phylogenetics.” In PCR Protocols: A Guide to Methods and Applications, edited by M. A. Innis , H. Gelfand , J. S. Sninsky , and T. J. White , 315–322. Academic Press. 10.1016/B978-0-12-372180-8.50042-1.

[gcb70885-bib-0087] Williams, C. J. R. 2017. “Climate Change in Chile: An Analysis of State‐Of‐The‐Art Observations, Satellite‐Derived Estimates and Climate Model Simulations.” Journal of Earth Science & Climatic Change 8: 400. 10.4172/2157-7617.1000400.

[gcb70885-bib-0088] Williams, J. P. , and J. E. Hallsworth . 2009. “Limits of Life in Hostile Environments: No Barriers to Biosphere Function?” Environmental Microbiology 11, no. 12: 3292–3308. 10.1111/j.1462-2920.2009.02079.x. 19840102 PMC2810447

[gcb70885-bib-0089] Williamson, B. , B. Tudzynski , P. Tudzynski , and J. A. L. Van Kan . 2007. “ *Botrytis cinerea*: The Cause of Grey Mould Disease.” Molecular Plant Pathology 8, no. 5: 561–580. 10.1111/j.1364-3703.2007.00417.x. 20507522

[gcb70885-bib-0090] World Health Organization . 2022. “WHO Fungal Priority Pathogens List to Guide Research, Development and Public Health Action.”

[gcb70885-bib-0091] Yde‐Andersen, A. 1980. “Infection Process and the Influence of Frost Damage in *Lachnellula willkommii*—A Literature Review.” European Journal of Forest Pathology 10, no. 1: 28–36. 10.1111/j.1439-0329.1980.tb00003.x.

[gcb70885-bib-0092] Yin, H. , T. X. Wang , Z. Y. Yang , et al. 2024. “ *Neocamarosporium betae* Causing Leaf Spot and Stem Necrosis Disease on *Chenopodium quinoa* in Shanxi Province, China.” Crop Protection 185: 106889. 10.1016/j.cropro.2024.106889.

