# Supplementary material for: Climate Change Projected to Double the Richness and Abundance of Soilborne Phytopathogenic Fungi in Southern Maritime Antarctica
Source: Glob Chang Biol. 2026 May 12;32:e70885. doi: 10.1111/gcb.70885 (PMC13162181; doi:10.1111/gcb.70885)
Supplement: Supplementary file 1 — Figure S1: Correlogram showing Spearman's correlation coefficients for associations between latitude and the climatic and edaphic factors used as predictor variables. Figure S2: Soil C/N ratio as a function of maximum air temperature of the warmest month (BIO5) across the latitudinal transect. Figure S3: The pH value of barren Maritime Antarctic soil treated with open top chambers for 5 years. Figure S4: Changes to climatic and edaphic factors across the latitudinal transect. Figure S5: Summary of data distributions. Figure S6: The amplicon sequence variant richness of soilborne plant and animal pathogenic fungi as functions of climatic and edaphic factors selected by LASSO regression. Figure S7: Principal component analysis of associations between climatic and edaphic factors and the abundances of pathogenic fungal genera. Figure S8: Soilborne plant and animal pathogenic fungal genera indicative of climatic and edaphic factors. Table S1: Genera assigned to plant or animal pathogen guilds. Table S2: Climatic and edaphic factors used as predictors and their median values along the transect. Table S3: Summary of the DNA sequencing data on which the analyses were based. Table S4: Intercepts and coefficients for predictors of the amplicon sequence variant richness of plant and animal pathogenic fungi derived from LASSO regression. Appendix S1: R script used to retrieve and analyse data. [file GCB-32-e70885-s001.docx]

**Table S1.** **Genera assigned to plant or animal pathogen guilds***

| **Genus** | **Guild** | **Reference** |
| --- | --- | --- |
| *Acremonium* | plant and animal | Mahamedi et al. (2025); Fernández-Silva et al. (2014) |
| *Akanthomyces* | animal | Xie et al. (2024) |
| *Alternaria* | plant and animal | Kodama (2019); Revankar and Sutton (2010) |
| *Annulohypoxylon* | plant | Gutiérrez-Flores et al. (2023) |
| *Antarctomyces* | animal | Brito Devoto et al. (2022) |
| *Aphanoascus* | animal | Yamaguchi et al*.* (2014) |
| *Arthroderma* | animal | Chen et al. (2023) |
| *Athelia* | plant | Tripathi et al. (2025) |
| *Atractiella* | plant | Allen et al. (2020) |
| *Aureobasidium* | animal | Revankar and Sutton (2010) |
| *Auxarthron* | animal | Deshmukh et al. (2018) |
| *Beauveria* | animal | Boomsma et al. (2014) |
| *Belonioscyphella* | plant | Egertová et al. (2016) |
| *Bergerella* | animal | Li et al. (2023) |
| *Botrytis* | plant | Dean et al. (2012) |
| *Cadophora* | plant | Llorens et al. (2025) |
| *Candida* | animal | World Health Organization (2022) |
| *Capronia* | animal | Teixeira et al. (2017) |
| *Ceratobasidium* | plant | Marelli et al. (2019) |
| *Chalara* | plant | Gross et al. (2014) |
| *Chrysosporium* | animal | Cabañes et al. (2014) |
| *Cladophialophora* | animal | Revankar and Sutton (2010) |
| *Cladosporium* | plant and animal | Thomma et al. (2005); Revankar and Sutton (2010) |
| *Coleophoma* | plant | Polashock et al. (2009) |
| *Comoclathris* | plant | Moral et al. (2017) |
| *Coniochaeta* | plant and animal | Damm et al. (2010); Kabtani et al. (2022) |
| *Cosmospora* | plant | Rosa et al. (2020) |
| *Cryptococcus* | animal | World Health Organization (2022) |
| *Cudoniella* | animal | Stoute et al. (2009) |
| *Cutaneotrichosporon* | animal | do Espírito Santo et al. (2020) |
| *Cyphellophora* | plant and animal | Gao et al. (2015); De Vries et al. (1986) |
| *Cystobasidium* | animal | Lu et al. (2023) |
| *Cytospora* | plant | Adams et al. (2006) |
| *Diaporthe* | plant | Gomes et al. (2013) |
| *Diatrype* | plant | Trouillas et al. (2010) |
| *Endosporium* | plant | Tsuneda et al. (2008) |
| *Entorrhiza* | plant | Bauer et al. (2015) |
| *Eocronartium* | plant | Davey et al. (2017) |
| *Epibryon* | plant | Haldeman and Greiff (2024) |
| *Exophiala* | animal | Revankar and Sutton (2010) |
| *Fusarium* | plant and animal | Dean et al. (2012); World Health Organisation (2022) |
| *Geomyces* | animal | Fisher et al. (2012) |
| *Gremmenia* | plant | Doğmuş-Lehtijärvi et al. (2016) |
| *Helminthosporium* | plant | Figueroa et al. (2018) |
| *Hemibeltrania*  **Table S1 (continued)** | plant | Rajeshkumar et al. (2016) |
| *Ilyonectria* | plant | Farh et al. (2018) |
| *Itersonilia* | plant | Pilkington et al. (2023) |
| *Knufia* | animal | Akimoto et al. (2025) |
| *Lachnellula* | plant | Sylvestreguinot and Delatour (1983) |
| *Lanzia* | plant | Brede (1991) |
| *Leptobacillium* | animal | Casique-Valdés et al. (2022) |
| *Leptosphaeria* | plant | Piątek, M. et al*.* (2020) |
| *Leucosporidium* | animal | Lu et al. (2023) |
| *Liangia* | animal | Wang et al. (2020) |
| *Malassezia* | animal | Hazen (1995) |
| *Microdochium* | plant | Hoshino (2025) |
| *Mucor* | animal | Ribes et al. (2000) |
| *Naganishia* | animal | Oliveira et al. (2021) |
| *Nectria* | plant | Silva et al. (2025) |
| *Neoascochyta* | plant | Ma et al. (2025) |
| *Neocamarosporium* | plant | Yin et al. (2024) |
| *Neophaeomoniella* | plant | van Dyk et al. (2021) |
| *Neosetophoma* | plant | Testempasis et al. (2024) |
| *Neostagonospora* | plant | Marin-Felix et al. (2019) |
| *Nigrograna* | animal | Santos et al. (2013) |
| *Ochroconis* | animal | Revankar and Sutton (2010) |
| *Octospora* | plant | Davey and Currah (2006) |
| *Ophiosphaerella* | plant | Hutchens et al. (2025) |
| *Papiliotrema* | animal | Bermann et al. (2025) |
| *Paraleptosphaeria* | plant | Piątek et al*.* (2020) |
| *Paraphaeosphaeria* | plant | Bi et al. (2023) |
| *Paraphoma* | plant | Lukina et al. (2025) |
| *Parastagonospora* | plant | Kariyawasam et al. (2023) |
| *Parathyridaria* | animal | Monpierre et al. (2025) |
| *Penicillium* | animal | Hazen (1995) |
| *Phaeococcomyces* | animal | De Hoog et al. (2003) |
| *Phaeosphaeria* | plant | McDonald et al. (2012) |
| *Pilidium* | plant | Torbati et al. (2019) |
| *Plectosphaerella* | plant | Li et al. (2025) |
| *Plenodomus* | plant | Razzaq et al. (2025) |
| *Pleurophoma* | plant | Behnke-Borowczyk et al. (2019) |
| *Pochonia* | animal | Hao et al. (2025) |
| *Protomyces* | plant | Leharwan et al. (2021) |
| *Pseudogymnoascus* | animal | Fisher et al. (2012) |
| *Pyrenochaeta* | plant and animal | Testen et al. (2023); Chabasse (2002) |
| *Pyrenochaetopsis* | plant | Sun et al. (2023) |
| *Pyrenophora* | plant | Murray and Brennan (2009) |
| *Rhinocladiella* | animal | Revankar and Sutton (2010) |
| *Schwanniomyces* | animal | Relich et al. (2016) |
| **Table S1 (continued)** |  |  |
| *Scytalidium* | plant and animal | Solís-García et al. (2021); Shah et al. (2025) |
| *Septoriella* | plant | Crous et al. (2015) |
| *Simplicillium* | animal | Yan et al. (2023) |
| *Sporormiella* | plant | Do Amaral et al. (2005) |
| *Stagonospora* | plant | Veselova et al. (2021) |
| *Stemphylium* | plant | Feng et al. (2025) |
| *Stilbella* | plant | Paim et al. (2012) |
| *Taphrina* | plant | Goldy et al. (2017) |
| *Thelebolus* | animal | Brito Devoto et al. (2022) |
| *Thyrostroma* | plant | Rasool et al. (2020) |
| *Tolypocladium* | animal | Ranout et al. (2024) |
| *Tranzscheliella* | plant | Li et al. (2017) |
| *Tricharina* | plant | Egger and Paden (1986) |
| *Trimmatostroma* | plant | Butin et al. (1996) |
| *Truncatella* | plant | Noshad et al. (2024) |
| *Venturia* | plant | Belete and Boyraz (2017) |

*Note that eight genera were assigned to both guilds

References cited in Table S1

Adams, G.C. et al. 2006. *Cytospora* species (*Ascomycota, Diaporthales, Valsaceae*): introduced and native pathogens of trees in South Africa. *Australasian Plant Pathology* 35: 521–548. https://doi.org/10.1071/AP06058

Akimoto, R. et al. 2025. A case of Chromoblastomycosis caused by *Knufia epidermidis* presenting as multiple dark brown papules in the nasal area. *Medical Mycology Journal* 66: 171–175. https://doi.org/10.3314/mmj.25-00015

Allen, R.N. et al. 2020. Red root rot of *Vicia sativa* caused by *Atractiella rhizophila*. *European Journal of Plant Pathology* 157: 293–297. https://doi.org/10.1007/s10658-020-01985-z

Bauer, R. et al. 2015. Entorrhizomycota: a new fungal phylum reveals new perspectives on the evolution of fungi. *PLoS One* 10: e0128183. https://doi.org/10.1371/journal.pone.0128183

Behnke-Borowczyk, J. et al. 2019. Fungi associated with *Cyclaneusma* needle cast in Scots pine in the west of Poland. *Forest Pathology* 49: e12487. https://doi.org/10.1111/efp.12487

Belete, T. and  Boyraz, N. 2017. Critical review on apple scab (*Venturia inaequalis*) biology, epidemiology, economic importance, management and defense mechanisms to the causal agent. *Journal of Plant Physiology and Pathology* 5: 1000166. https://doi.org/10.4172/2329-955X.1000166

Bermann, C. et al. 2025. Exploring virulence attributes in non-*Cryptococcus* Tremellomycetes. *Medical Mycology* 63: myaf086. https://doi.org/10.1093/mmy/myaf086

Bi, Y.M. et al. 2023. The relationship between shifts in the rhizosphere microbial community and root rot disease in a continuous cropping American ginseng system. *Frontiers in Microbiology* 14: 1097742 https://doi.org/10.3389/fmicb.2023.1097742

Boomsma, J.J. et al. 2014. Evolutionary interaction networks of insect pathogenic fungi. *Annual Review of Entomology* 59: 467–485. https://doi.org/10.1146/annurev-ento-011613-162054

Brede, A.D. 1991. Interaction of management factors on dollar spot disease severity in tall fescue turf. *Hortscience* 26: 1391–1392. https://doi.org/10.21273/HORTSCI.26.11.1391

Brito Devoto, T. et al. 2022. Exploring fungal diversity in Antarctic wildlife: isolation and molecular identification of culturable fungi from penguins and pinnipeds. *New Zealand Veterinary Journal* 70: 263–272. https://doi.org/10.1080/00480169.2022.2087784

Butin, H. et al*.* 1996. *Trimmatostroma abietis* sp. nov. (hyphomycetes) and related species. *Antonie van Leeuwenhoek* 69: 203–209. https://doi.org/10.1007/BF00399607

Cabañes, F.J. et al. 2014. *Chrysosporium*-related fungi and reptiles: a fatal attraction. *PLoS Pathogens* 10: e1004367. https://doi.org/10.1371/journal.ppat.1004367

Casique-Valdés, R. et al. 2022. New records of fungal pathogens of invertebrates from endemic pine forests in Mexico. *Check List* 18: 67–77. https://doi.org/10.15560/18.1.67

Chabasse D. 2002. Phaeohyphomycetes agents of phaeohyphomycosis: emerging fungi. *Journal de Mycologie Médicale* 12: 65–85.

Chen, Q. et al. 2023. Zoonotic fungus *Arthroderma multifidum* causing chronic pulmonary infection.

*International Journal of Infectious Diseases* 130: 17–19. https://doi.org/10.1016/j.ijid.2023.02.010

Crous, P.W. et al. 2015. The Genera of Fungi - fixing the application of the type species of generic names - G 2: *Allantophomopsis, Latorua, Macrodiplodiopsis, Macrohilum, Milospium, Protostegia, Pyricularia, Robillarda, Rotula, Septoriella, Torula*, and *Wojnowicia*. *IMA Fungus* 6: 163–198. https://doi.org/10.5598/imafungus.2015.06.01.11

Damm, U. et al. 2010. *Coniochaeta* (*Lecythophora*), *Collophora* gen. nov and *Phaeomoniella* species associated with wood necroses of *Prunus* trees. *Persoonia* 24: 60–80. https://doi.org/10.3767/003158510X500705

Davey, M.L. and Currah, R.S. 2006. Interactions between mosses (Bryophyta) and fungi. *Canadian Journal of Botany* 84: 1509–1519. https://doi.org/10.1139/b06-120

Davey, M.L. et al. 2017. Host and tissue variations overshadow the response of boreal moss-associated fungal communities to increased nitrogen load. *Molecular Ecology* 26: 571–588. https://doi.org/10.1111/mec.13938

Dean, R. et al. 2012. The top 10 fungal pathogens in molecular plant pathology. *Molecular Plant Pathology* 13: 414–430. https://doi.org/10.1111/j.1364-3703.2011.00783.x

Deshmukh, S.K. et al. 2018. Keratinophilic fungi from the vicinity of salt pan soils of Sambhar lake Rajasthan (India). *Journal de Mycologie Médicale* 28: 457–461. https://doi.org/10.1016/j.mycmed.2018.06.002

de Hoog, G.S. et al. 2003. Species diversity and polymorphism in the *Exophiala spinifera* clade containing opportunistic black yeast-like fungi. *Journal of Clinical Microbiology* 41: https://doi.org/10.1128/jcm.41.10.4767-4778.2003

De Vries, G.A. et al. 1986. Description of *Cyphellophora pluriseptata* sp. nov. *Antonie van Leeuwenhoek* 52: 141–143. https://doi.org/10.1007/BF00429317

Do Amaral, A.L. et al. 2005. Pathogenic fungi causing symptoms similar to *Phaeosphaeria* leaf spot of maize in Brazil. *Plant Disease* 89: 44–49. https://doi: 10.1094/PD-89-0044

do Espírito Santo, E.P.T. et al*.* 2020. Molecular identification, genotyping, phenotyping, and antifungal susceptibilities of medically important *Trichosporon*, *Apiotrichum*, and *Cutaneotrichosporon* species. *Mycopathologia* 185: 307–317. https://doi.org/10.1007/s11046-019-00407-x

Doğmuş-Lehtijärvi, H.T. et al. 2016. Impacts of inoculation with *Herpotrichia pinetorum, Gremmenia infestans* and *Gremmeniella abietina* on *Pinus nigra* subsp. *pallasiana* and *Cedrus libani* seedlings in the field. *Forest Pathology* 46: 47–53. https://doi.org/10.1111/efp.12213

Egertová, Z. et al. 2016. *Belonioscyphella hypnorum* (Helotiales), a rarely reported bryoparasitic ascomycete new for the Czech Republic. *Ascomycete.org* 8: 91–95. https://doi.org/10.25664/art-0176

Egger, K.N. and Paden, J.W. 1986. Biotrophic associations between lodgepole pine seedlings and postfire ascomycetes (Pezizales) in monoxenic culture. *Canadian Journal of Botany* 64: 2719–2725. https://doi.org/10.1139/b86-359

Farh, M.E.-A. et al. 2018. *Cylindrocarpon destructans*/*Ilyonectria radicicola*-species complex: causative agent of ginseng root-rot disease and rusty symptoms. *Journal of Ginseng Research* 42: 9–15. https://doi.org/10.1016/j.jgr.2017.01.004

Feng, H. et al*.* 2025. *Alternaria* and *Stemphylium* species associated with leaf blight and fruit rot of field-grown tomatoes in Australia. *European Journal of Plant Pathology*. https://doi.org/10.1007/s10658-025-03163-5

Fernández-Silva, F. et al. 2014. Experimental murine acremoniosis: an emerging opportunistic human infection. *Medical Mycology* 52: 29–35. https://doi.org/10.3109/13693786.2013.797610

Figueroa, M. et al. 2018. A review of wheat diseases—a field perspective. *Molecular Plant Pathology* 19: 1523–1536. https://doi.org/10.1111/mpp.12618

Fisher, M. et al. 2012. Emerging fungal threats to animal, plant and ecosystem health. *Nature* 484: 186–194. https://doi.org/10.1038/nature10947

Gao, L. et al. 2015. Three new species of *Cyphellophora* (Chaetothyriales) associated with sooty blotch and flyspeck. *PLoS ONE* 10: e0136857. https://doi.org/10.1371/journal.pone.0136857

Goldy, C. et al. 2017. Comparative proteomic and metabolomic studies between *Prunus persica* genotypes resistant and susceptible to *Taphrina deformans* suggest a molecular basis of resistance. *Plant Physiology and Biochemistry* 118: 245–255. https://doi.org/10.1016/j.plaphy.2017.06.022

Gomes, R.R. et al. 2013. *Diaporthe*: a genus of endophytic, saprobic and plant pathogenic fungi. *Persoonia* 31: 1–41. http://dx.doi.org/10.3767/003158513X666844

Gross, A. et al. 2014. *Hymenoscyphus pseudoalbidus*, the causal agent of European ash dieback. *Molecular Plant Pathology* 15: 5–21. https://doi.org/10.1111/mpp.12073

Gutiérrez-Flores, L.M. et al. 2023. Potential use of bacterial strain in the genus *Serratia* to biocontrol fungal pathogens associated with defoliation on *Pinus patula*. *Biocontrol Science and Technology* 33: 640–653. https://doi.org/10.1080/09583157.2023.2210276

Haldeman, M. and Greiff, G. 2024. *Epibryon endocarpum* Döbbeler, a little-known hepaticolous ascomycete new to North America and Britain. *Ascomycete.org* 16: 11–14. https://doi.org/10.25664/art-0385

Hao, L. et al. 2025. Dynamic proteomic changes and ultrastructural insights into *Pochonia chlamydosporia*’s parasitism of *Parascaris equorum* eggs. *Frontiers in Cellular and Infection Microbiology* 15: 1600620. https://doi.org/10.3389/fcimb.2025.1600620

Hazen, K.C. 1995. New and emerging yeast pathogens. *Clinical Microbiology Reviews* 8: 462–478. https://doi.org/10.1128/CMR.8.4.462

Hoshino, T. 2025. Diversity, geographical distribution and environmental adaptations of snow molds. *Mycoscience* 66: 334–349. https://doi.org/10.47371/mycosci.2025.09.001

Hutchens, W.J. et al. 2025. Fifteen years of findings: advancements in spring dead spot research from 2009 to 2024. *Crop Science* 65: e21367. https://doi.org/10.1002/csc2.21367

Kabtani, J. et al. 2022. *Coniochaeta massiliensis* sp. nov. isolated from a clinical sample. *Journal of Fungi* 8: 999. https://doi.org/10.3390/jof8100999

Kariyawasam, G.K. et al. 2023. The necrotrophic pathogen *Parastagonospora nodorum* is a master manipulator of wheat defense. *Molecular Plant-Microbe Interactions* 12: 764–773. https://doi.org/10.1094/MPMI-05-23-0067-IRW

Kodama, M. 2019. Evolution of pathogenicity in *Alternaria* plant pathogens. *Journal of General Plant Pathology* 85: 471–474. https://doi.org/10.1007/s10327-019-00877-3

Leharwan, M. et al. 2021. Characterization of *Protomyces macrosporus* causing stem gall of coriander (*Coriandrum sativum*). *The Indian Journal of Agricultural Sciences* 91: 1561–1565.

https://doi.org/10.56093/ijas.v91i11.118529

Li, Y. et al*.* 2023. Characteristics of the nasal mucosa of commercial pigs during normal development. *Veterinary Research* 54: 37. https://doi.org/10.1186/s13567-023-01164-y

Li, Y. et al. 2025. First report of *Plectosphaerella cucumerina* causing wilt in cucumber (*Cucumis sativus*) in China. *Plant Disease* 109: 1787. https://doi.org/10.1094/PDIS-02-25-0344-PDN

Li, Y.M. et al. 2017. Cryptic diversity in *Tranzscheliella* spp. (*Ustilaginales*) is driven by host switches. *Scientific Reports* 7: 43549. https://doi.org/10.1038/srep43549

Llorens, E. et al. 2025. Interaction among *Cadophora luteo-olivacea*, *Phaeoacremonium minimum*, and *Pseudophaeomoniella* *oleicola* in olive multi-infections and cultivar susceptibility. *Plant Disease* 109: 2147–2153. https://doi.org/10.1094/PDIS-11-24-2466-RE

Lu, S. et al. 2023. Effects of *Cryptosporidium parvum* infection on intestinal fungal microbiota in yaks (*Bos grunniens*). *Microbial Pathogenesis* 183: 106322. https://doi.org/10.1016/j.micpath.2023.106322

Lukina, E. et al. 2025. *Paraphoma rubrobrunnea* sp. nov. and *Paraphoma cirsii* sp. nov. from *Cirsium* species and biological characterization of their phytotoxins. *Mycological Progress* 24: 69. https://doi.org/10.1007/s11557-025-02077-0

Ma, L. et al. 2025. First report of *Neoascochyta cylindrispora* causing leaf spot on *Taraxacum mongolicum* in China. *Plant Disease* 109: 2593. https://doi.org/10.1094/PDIS-12-24-2629-PDN

Mahamedi, A.E. et al. 2025. *Acremonium quercivorum* (*Hypocreales, Sordariomycetes*), a new species from oak (*Quercus* spp.) growing in Algeria. *Mycobiology* 53: 717–724. https://doi.org/10.1080/12298093.2025.2550068

Marelli, J.-P. et al. 2019. Chocolate under threat from old and new Cacao diseases. *Phytopathology* 109: 1331–1343. https://doi.org/10.1094/PHYTO-12-18-0477-RVW

Marin-Felix, Y. et al. 2019. Genera of phytopathogenic fungi: GOPHY 3. *Studies in Mycology* 94: 1–124. https://doi.org/10.1016/j.simyco.2019.05.001

McDonald, M.C. et al. 2012. Phylogenetic and population genetic analyses of *Phaeosphaeria nodorum* and its close relatives indicate cryptic species and an origin in the Fertile Crescent. *Fungal Genetics and Biology* 49: 882–895. https://doi.org/10.1016/j.fgb.2012.08.001

Monpierre, L. et al. 2025. Contribution of shotgun metagenomics in the diagnosis of a subcutaneous phaeohyphomycosis caused by *Parathyridaria percutanea*. *British Journal of Dermatology* 192: 948–950. https://doi.org/10.1093/bjd/ljaf018

Moral, J. et al. 2017. [Identification of fungal species associated with branch dieback of olive and resistance of table cultivars to *Neofusicoccum mediterraneum* and *Botryosphaeria dothidea*](https://apsjournals.apsnet.org/doi/full/10.1094/PDIS-06-16-0806-RE). *Plant Pathology* 101: 306–316. http://dx.doi.org/10.1094/PDIS-06-16-0806-RE

Murray, G.M. and Brennan, J.P. 2009. Estimating disease losses to the Australian wheat industry. *Australasian Plant Pathology* 38: 558–570. https://doi.org/10.1071/AP09053

Noshad, D. et al. 2024. First report of *Truncatella angustata* causing leaf blight on *Thuja plicata* in Canada. *Plant Disease* 108: 785. https://doi.org/10.1094/PDIS-03-23-0471-PDN

Oliveira, L.S.d.S. et al. 2021. Comparison of *Cryptococcus gattii*/*neoformans* species complex to related genera (*Papiliotrema* and *Naganishia*) reveal variances in virulence associated factors and antifungal susceptibility. *Frontiers in Cellular and Infection Microbiology* 11: 642658. https://doi.org/10.3389/fcimb.2021.642658

Paim, E.C.A. et al. 2012. Etiologia do declínio de mangostanzeiros no sul da Bahia. *Revista Brasileira de Fruticultura* 34: 1074–1083. https://doi.org/10.1590/S0100-29452012000400014

Piątek, M. et al*.* 2020. Phylogenetic placement of *Leptosphaeria polylepidis*, a pathogen of Andean endemic *Polylepis tarapacana*, and its newly discovered mycoparasite *Sajamaea mycophila* gen. et sp. nov.. *Mycological Progress* 19: 1–14. https://doi.org/10.1007/s11557-019-01535-w

Pilkington, S. et al. 2023. Confirmation of *Itersonilia perplexans* infecting Pyrethrum (*Tanacetum cinerariifolium*) in Australia. *Plant Disease* 107: 2258. https://doi.org/10.1094/PDIS-11-22-2604-PDN

Polashock, J.J. et al. 2009. The North American cranberry fruit rot fungal community: a systematic overview using morphological and phylogenetic affinities. *Plant Pathology* 58: 1116–1127.

<https://doi.org/10.1111/j.1365-3059.2009.02120.x>

Pointing, S. B., Y. Chan, D. C. Lacap, M. C. Y. Lau, J. A. Jurgens, and R. L. Farrell. 2009.“Highly Specialized Microbial Diversity in Hyper‐Arid Polar Desert.” Proceedings of theNational Academy of Sciences of the United States of America 106, no. 47: 19964–19969. .

Rajeshkumar, K.C. et al. 2016. Taxonomic re-evaluation and phylogenetic position of *Hemibeltrania cinnamomi* within Xylariales. *Mycotaxon* 131: 87–94. http://dx.doi.org/10.5248/131.87

Ranout, A.S. et al. 2024. Pathogenicity and compatibility studies of native *Tolypocladium inflatum* and *Clonostachys krabiensis* against *Tetranychus urticae*. *Journal of Applied Entomology* 148: 1210–1222.

https://doi.org/10.1111/jen.13339

Rasool, R.S. et al. 2020. *Thyrostroma carpophilum* insertional mutagenesis: a step towards understanding its pathogenicity mechanism. *Journal of Microbiological Methods* 171: 105885. https://doi.org/10.1016/j.mimet.2020.105885

Razzaq, K. et al. 2025. Integrated management strategies for blackleg disease of canola amidst climate change challenges. *Journal of Fungi* 11: 514. https://doi.org/10.3390/jof11070514

Relich, R.F. et al. 2016. *Schwanniomyces etchellsii*: an unusual cause of fungemia in a patient with cholecystitis. *Diagnostic Microbiology and Infectious Disease* 84: 221–222. https://doi.org/10.1016/j.diagmicrobio.2015.11.010

Revankar, S.G. and Sutton, D.A. 2010. Melanized fungi in human disease. *Clinical Microbiology Reviews* 23: 884–928. https://doi.org/10.1128/cmr.00019-10

Ribes, J.A. et al. 2000. Zygomycetes in human disease. *Clinical Microbiology Reviews* 13: 236–301. https://doi.org/10.1128/cmr.13.2.236

Rosa, L.H. et al. 2020. Opportunistic fungi found in fairy rings are present on different moss species in the Antarctic Peninsula. *Polar Biology* 43: 587–596. https://doi.org/10.1007/s00300-020-02663-w

Santos, D.W.C.L. et al. 2013. Molecular identification of melanised non-sporulating moulds: a useful tool for studying the epidemiology of phaeohyphomycosis. *Mycopathologia* 175: 445–454. https://doi.org/10.1007/s11046-012-9608-x

Shah, A.A. et al. 2025. Unveiling onychomycosis: pathogenesis, diagnosis, and innovative treatment strategies. *Microbial Pathogenesis* 198: 107111. https://doi.org/10.1016/j.micpath.2024.107111

Silva, T.F. et al. (2025). Four new fungal pathogens causing avocado dieback in Brazil. *Crop Protection* 192: 107168. https://doi.org/10.1016/j.cropro.2025.107168

Solís-García, I.A. et al. 2021. *Phytophthora* root rot modifies the composition of the avocado rhizosphere microbiome and increases the abundance of opportunistic fungal pathogens. *Frontiers in Microbiology* 11: 574110. https://doi.org/10.3389/fmicb.2020.574110

Stoute, S.T. et al. 2009. Mycotic pododermatitis and mycotic pneumonia in commercial turkey poults in Northern California. *Journal of Veterinary Diagnostic Investigation* 21: 554–557. https://doi.org/10.1177/104063870902100424

Sun, Q. et al. 2023. Continuous wheat/soybean cropping influences soybean yield and rhizosphere microbial community structure and function. *Agronomy* 13: 28. https://doi.org/10.3390/agronomy13010028

Sylvestreguinot, G. and Delatour, C. 1983. Feasibility of evaluating the susceptibility of *Larix* to *Lachnellula wilkommii* by artificial inoculation. *Anneles des Sciences Forestieres* 40: 337–354. http://dx.doi.org/10.1051/forest:19830402

Teixeira, M.M. et al. 2017. Exploring the genomic diversity of black yeasts and relatives (*Chaetothyriales*, *Ascomycota*). *Studies in Mycology* 86: 1–28. http://dx.doi.org/10.1016/j.simyco.2017.01.001

Testempasis, S.I. et al. 2024. Grapevine trunk diseases in Greece: disease incidence and fungi involved in discrete geographical zones and varieties. *Journal of Fungi* 10: 2. https://doi.org/10.3390/jof10010002

Testen, A.L. et al. 2023. A quantitative PCR method to detect the tomato corky root rot pathogen, *Pyrenochaeta lycopersici* and *P. terrestris*. *Plant Disease* 107: 2673–2678. https://doi.org/10.1094/PDIS-08-22-2009-RE

Thomma, B.P.H.J. et al. 2005. *Cladosporium fulvum* (syn. *Passalora fulva*), a highly specialized plant pathogen as a model for functional studies on plant pathogenic Mycosphaerellaceae. *Molecular Plant Pathology* 6: 379–393. https://doi.org/10.1111/j.1364-3703.2005.00292.x

Torbati, M. et al. 2019. Occurrence of fruit rot on cornelian cherry caused by *Pilidium lythri* in Iran.

*Crop Protection* 125: 104884. https://doi.org/10.1016/j.cropro.2019.104884

Tripathi, S. et al. 2025. Field evaluation of peanut cultivars to early leaf spot, late leaf spot, and southern blight at Raymond, MS, 2023. *Plant Health Progress* 26: 722. https://doi.org/10.1094/PHP-01-25-0035-PDMR

Trouillas, F. P. et al. 2010. Diversity of diatrypaceous fungi associated with grapevine canker diseases in California. *Mycologia* 102: 319–336. https://doi.org/10.3852/08-185

Tsuneda, A. et al. 2008. *Endosporium*, a new endoconidial genus allied to the Myriangiales. *Canadian Journal of Botany* 86: 1020–1032. https://doi.org/10.1139/B08-054

van Dyk, M. et al. 2021. Pathogenicity testing of fungal isolates associated with olive trunk diseases in South Africa. *Plant Disease* 105: 4060–4073. https://doi.org/10.1094/PDIS-08-20-1837-RE

Veselova, S.V. et al. 2021. Ethylene-cytokinin interaction determines early defense response of wheat against *Stagonospora nodorum* Berk. *Biomolecules* 11: 174. https://doi.org/10.3390/biom11020174

Wang, YB. et al*.* 2020. Multigene phylogeny of the family Cordycipitaceae (Hypocreales): new taxa and the new systematic position of the Chinese cordycipitoid fungus *Paecilomyces hepiali*. *Fungal Diversity* 103: 1–46. https://doi.org/10.1007/s13225-020-00457-3

World Health Organization 2022. WHO fungal priority pathogens list to guide research, development and

public health action. Geneva. ISBN 978-92-4-006024-1

Xie, W. et al. 2024. *Akanthomyces attenuatum* infection and effects on the reproduction of *Frankliniella occidentalis.* *Biocontrol Science and Technology* 34: 1006–1019. https://doi.org/10.1080/09583157.2024.2396981

Yamaguchi, S. et al*.* 2014. Isolation of dermatophytes and related species from domestic fowl (*Gallus gallus domesticus*). *Mycopathologia* 178: 135–143. https://doi.org/10.1007/s11046-014-9758-0

Yan, Q.H. et al. 2023. *Simplicillium sinense* sp. nov., a novel potential pathogen of tinea faciei. *Frontiers in Microbiology* 14: 1156027. https://doi.org/10.3389/fmicb.2023.1156027

Yin, H. et al. 2024. *Neocamarosporium betae* causing leaf spot and stem necrosis disease on *Chenopodium quinoa* in Shanxi Province, China. *Crop Protection* 185: 106889. https://doi.org/10.1016/j.cropro.2024.106889

**Table S2. Climatic and edaphic factors used as predictors and their median values along the transect**

| Factor | Median value^*^ |
| --- | --- |
| *Climatic* |  |
| BIO1 (mean annual air temperature) | -0.75 °C |
| BIO4 (air temperature seasonality)^**^ | 276.6 |
| BIO5 (maximum air temperature of the warmest month) | 3.35 °C |
| BIO6 (minimum air temperature of the coldest month) | -6.15 °C |
| BIO7 (annual range in air temperature) | 12.3 °C |
| BIO12 (annual precipitation) | 1.39 m yr^-1^ |
| BIO15 (precipitation seasonality)^†^ | 14.6 |
|  |  |
| *Edaphic* |  |
| pH value | 5.88 |
| C/N ratio | 8.21 |
| NO_3_^-^-N concentration (mg L^-1^) | 0.03 |
| NH_4_^-^-N concentration (mg L^-1^) | 0.07 |
| Total dissolved nitrogen (mg L^-1^) | 2.62 |
| Total dissolved organic carbon (mg L^-1^) | 25.07 |
| ^*^ Note that median values were used as the cut-off points in  indicator analyses  ^**^standard deviation × 100  ^†^coefficient of variation |  |

**Table S3.** **Summary of the DNA sequencing data on which the analyses were based**

| Parameter | Total | Minimum per sample | Mean per sample | Maximum per sample |
| --- | --- | --- | --- | --- |
| Number of DNA reads | 1,532,055 | 5,918 | 26,878 | 62,883 |
|  |  |  |  |  |
| *Plant pathogens* |  |  |  |  |
| Number of genera | 67 |  |  |  |
| Number of ASVs | 189 |  |  |  |
| Number of OTUs | 125 | 0 | 7.8 | 18.0 |
| Relative abundance (%) |  | 0 | 12.4 | 67.3 |
|  |  |  |  |  |
| *Animal pathogens* |  |  |  |  |
| Number of genera | 46 |  |  |  |
| Number of ASVs | 159 |  |  |  |
| Number of OTUs | 106 | 0 | 5.7 | 16.0 |
| Relative abundance (%) |  | 0 | 10.5 | 51.1 |

Note that data for plant and animal pathogens include eight genera, 35 ASVs and 19 OTUs that were assigned to both guilds

**Table S4.** I**ntercepts and coefficients for predictors of the amplicon sequence**

**variant richness of plant and animal pathogenic fungi derived from LASSO**

**regression**

|  |  | Plant pathogenic fungi Animal pathogenic fungi | | |
| --- | --- | --- | --- | --- |
|  |  | Richness  (no. ASVs) |  | Richness  (no. ASVs) |
| Intercept |  | 3.309 × 10^0^ |  | 6.764 × 10^0^ |
| BIO1 |  | 6.907 × 10^-1 ***^ |  | 1.680 × 10^-1 ***^ |
| BIO4 |  | 0 |  | 0 |
| BIO5 |  | 1.035 × 10^-3^ |  | 9.769 × 10^-2^ |
| BIO6 |  | 0 |  | 0 |
| BIO7 |  | 0 |  | 0 |
| BIO12 |  | 0 |  | -6.876 × 10^-1 ***^ |
| BIO15 |  | 0 |  | 0 |
| pH value |  | 0.947 × 10^-1 *^ |  | 6.970 × 10^-2^ |
| C/N ratio |  | 0 |  | 0 |
| NO_3_^-^-N |  | 0 |  | 0 |
| NH_4_^+^-N |  | -1.208 × 10^-2^ |  | 0 |
| TDN |  | 0 |  | 0 |
| TDOC |  | 0 |  | 0 |

Data shown are intercepts and coefficients with λ set to 1.952 × 10^-1^ and 2.744 × 10^-1^

for the amplicon sequence variant richness of plant and animal pathogenic fungi,

respectively. Asterisks denote the significance of predictors derived from analysis

of variance (*; *P*<0.05 and ***; *P*<0.001). *Abbreviations*: ASVs, amplicon sequence

variants; TDN, total dissolved nitrogen; TDOC, total dissolved organic carbon.


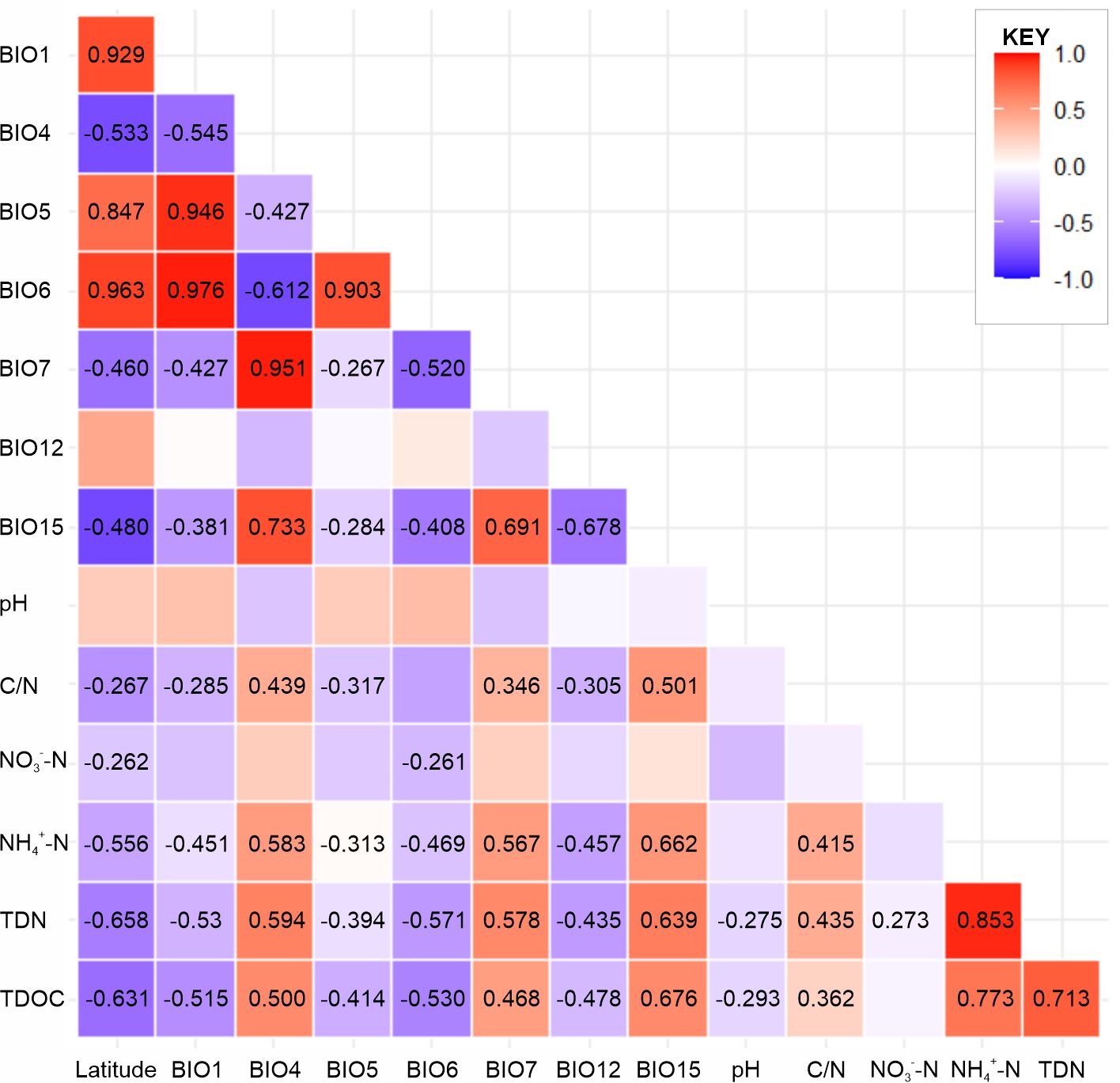


**Figure S1 |** **Correlogram showing Spearman’s correlation coefficients for associations between latitude and the climatic and edaphic factors used as predictor variables.** Values are statistically significant (*P*<0.05) coefficients, with colours denoting coefficient values (see key).

**
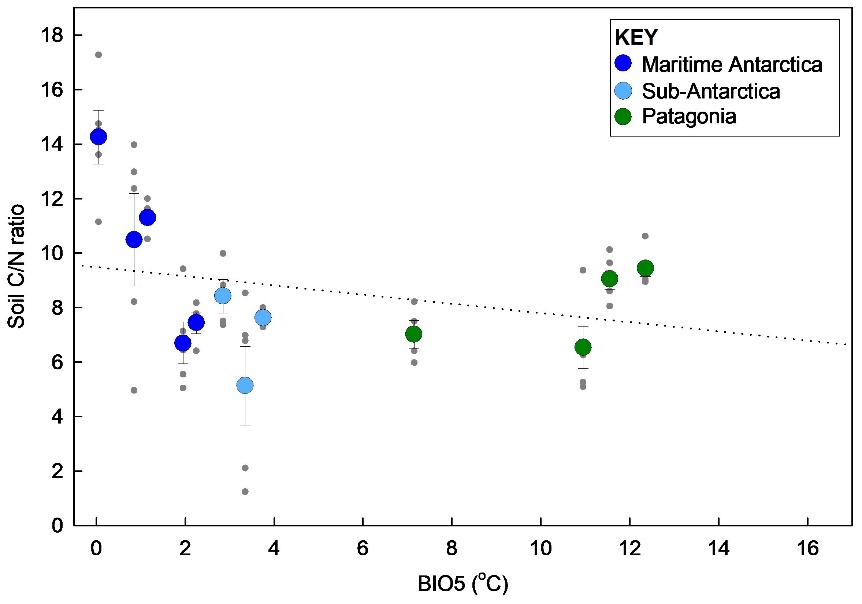
**

**Figure S2 | Soil C/N ratio as a function of maximum air temperature of the warmest month (BIO5) across the latitudinal transect.** Sampling sites are colour-coded for geographical region (see key). The dotted line shows the linear regression fit, indicating a -0.169 unit reduction in soil C/N ratio for each degree Celsius rise in BIO5.

**
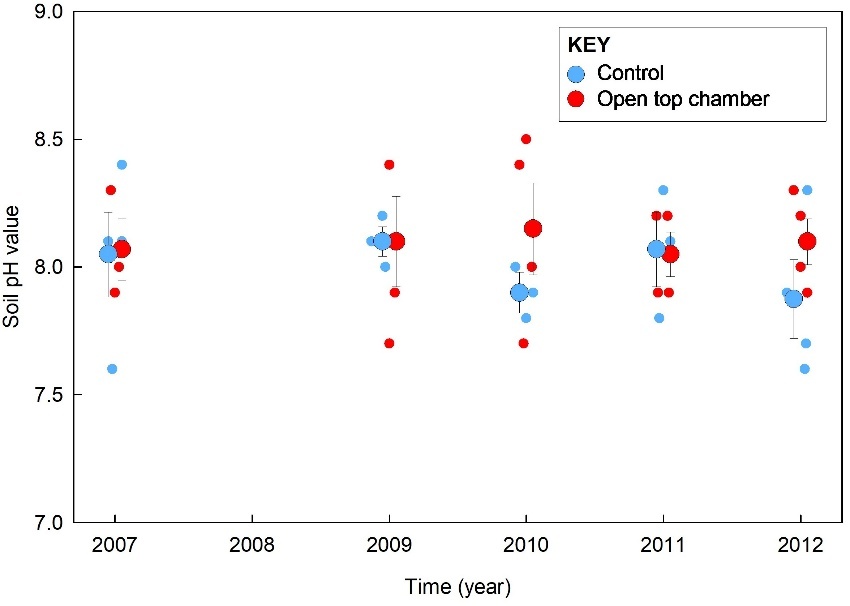
**

**Figure S3 | The pH value of barren Maritime Antarctic soil treated with open top chambers for five years.** Mean soil pH value ± SEM in open top chambers (red circles) and in control plots outside chambers (blue circles) are shown along with individual data points. Note that chambers, which increased mean annual soil surface temperature by 1 °C, were placed over soil in 2007. Soil pH value was measured in water. Student’s *t*-tests indicated no significant (*P*<0.05) effects of the treatment on the pH value of soil at each sampling. Data are from the experiment reported by Newsham et al. (2022).


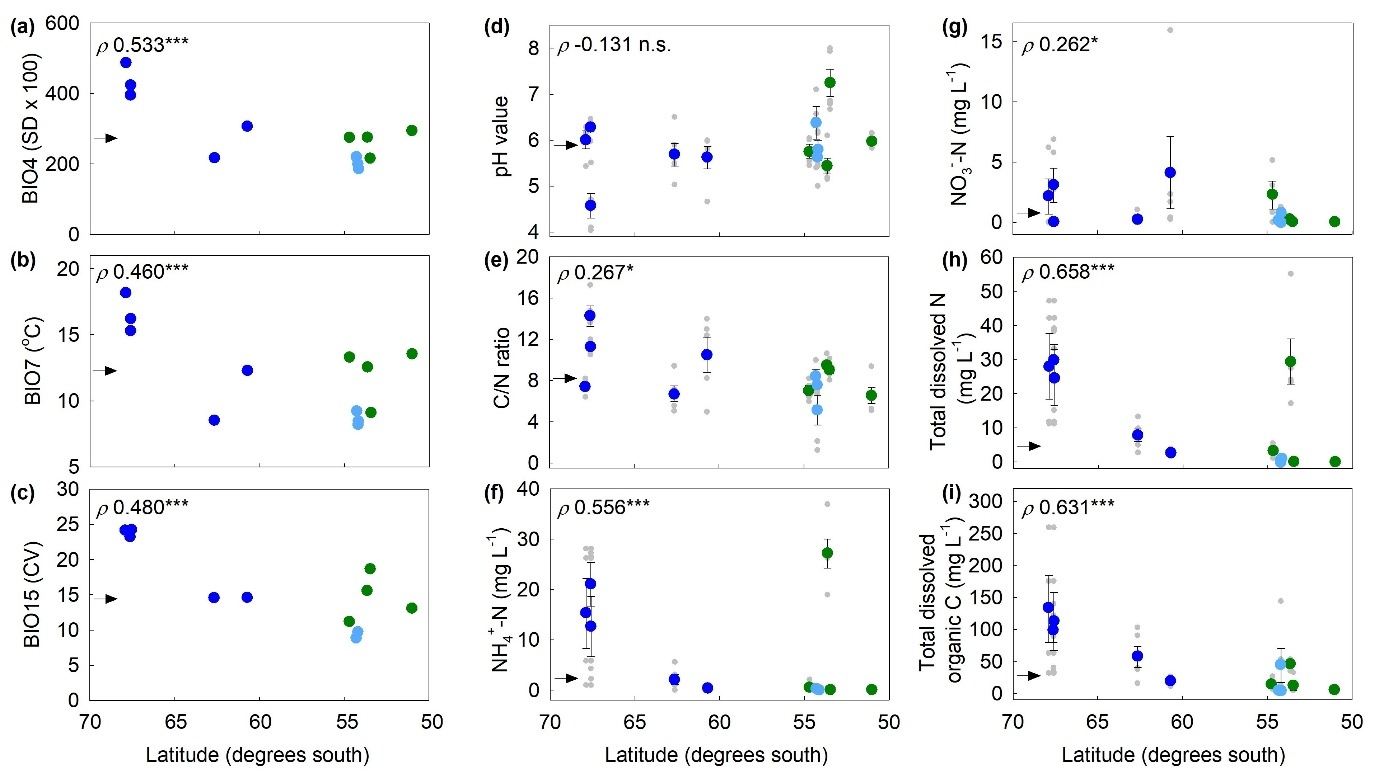


**Figure S4** | **Changes to climatic and edaphic factors across the latitudinal transect.** Spearman’s rank correlation coefficients (*ρ*) are shown, with asterisks denoting statistical significance (*, *P*<0.05 and ***, *P*<0.001). Arrows on *y*-axes indicate the median value of each factor. Sampling sites are colour-coded for geographical region (see key in Figure S2). *Abbreviations*: SD, standard deviation; CV, coefficient of variation; n.s., not significant.

**
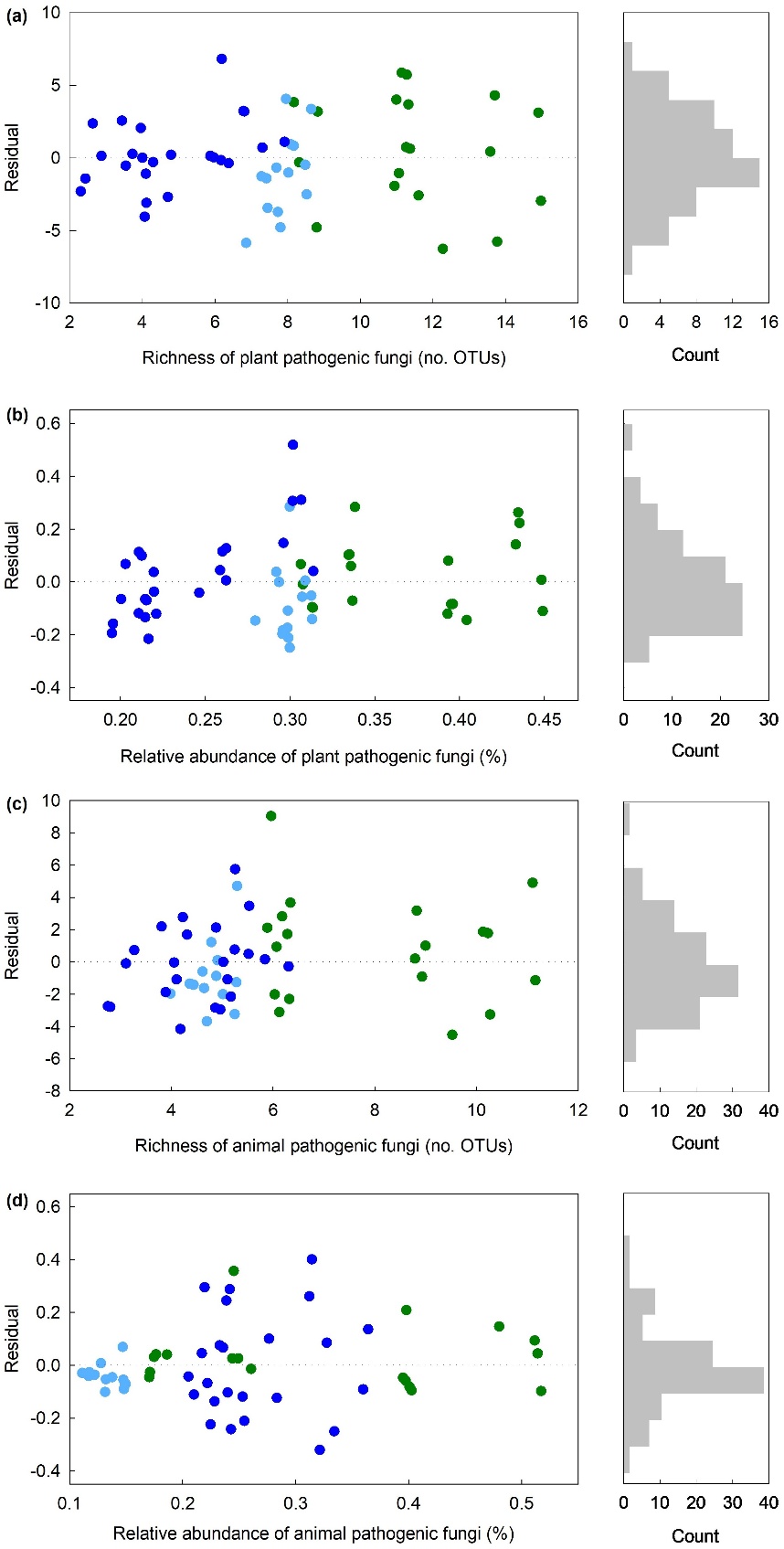
**

**Figure S5 | Summary of data distributions.** Residual plots (left column) and histograms (right column) for (a) the OTU richness and (b) the relative abundance of plant pathogenic fungi and (c) the OTU richness and (d) the relative abundance of animal pathogenic fungi. Relative abundance data were Hellinger-transformed prior to analyses. Sampling sites are colour-coded for geographical region (see key in Figure S2).


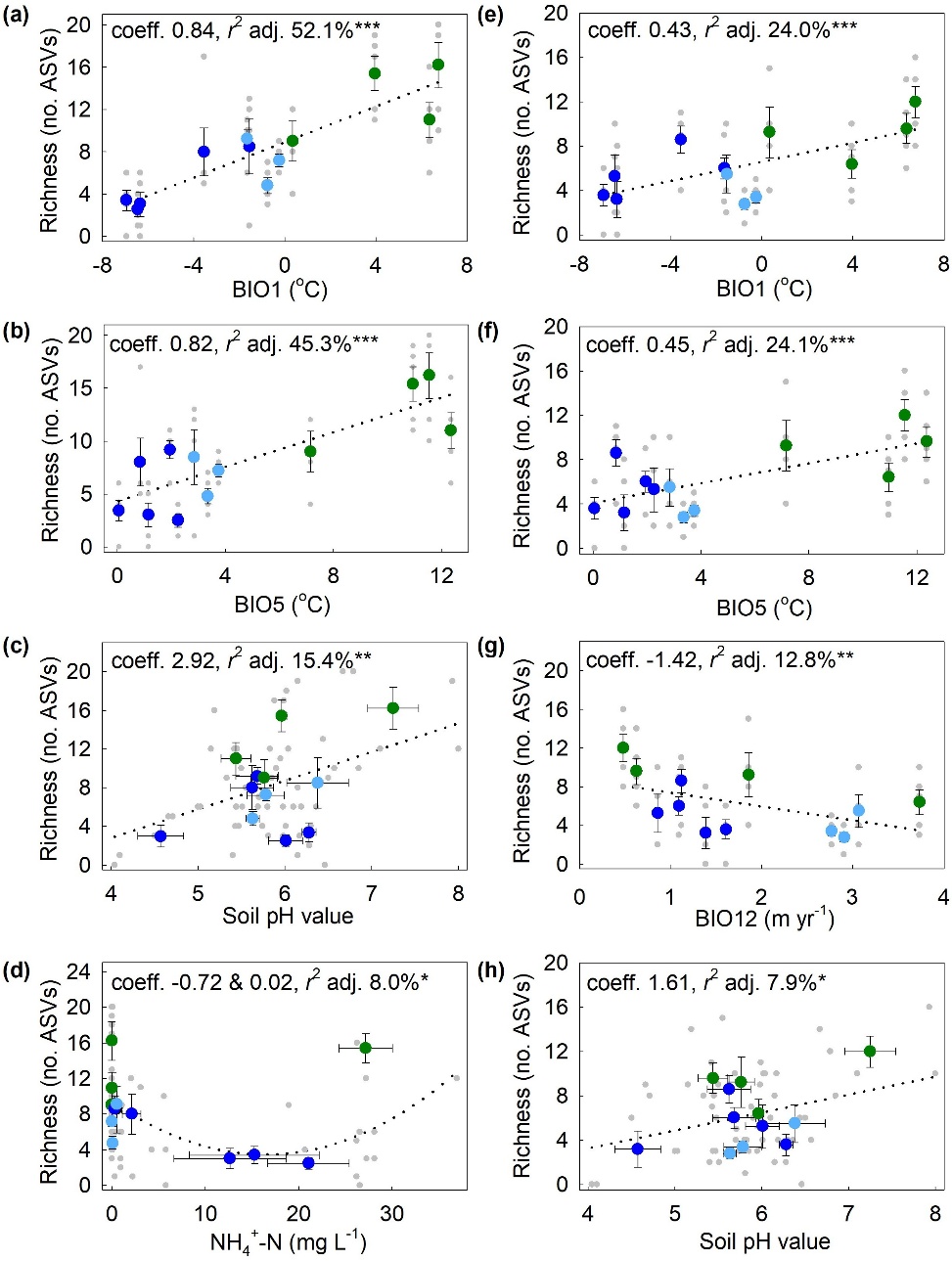


**Figure S6** | **The amplicon sequence variant richness of soilborne plant and animal pathogenic fungi as functions of climatic and edaphic factors selected by LASSO regression.** (a–d) Amplicon sequence variant richness of plant pathogenic fungi as functions of BIO1, BIO5, soil pH value and NH_4_^+^-N concentration. (e–h) Amplicon sequence variant richness of animal pathogenic fungi as functions of BIO1, BIO5, BIO12 and soil pH value. Values are means ± SEM and grey dots show individual data points. Dotted lines show linear or quadratic fits from regression models. The first and second coefficients shown in panel d are linear and squared components, respectively. Sampling sites are colour-coded for geographical region (see key in Figure S2). *Abbreviations*: ASVs, amplicon sequence variants; coeff., coefficient; adj., adjusted.


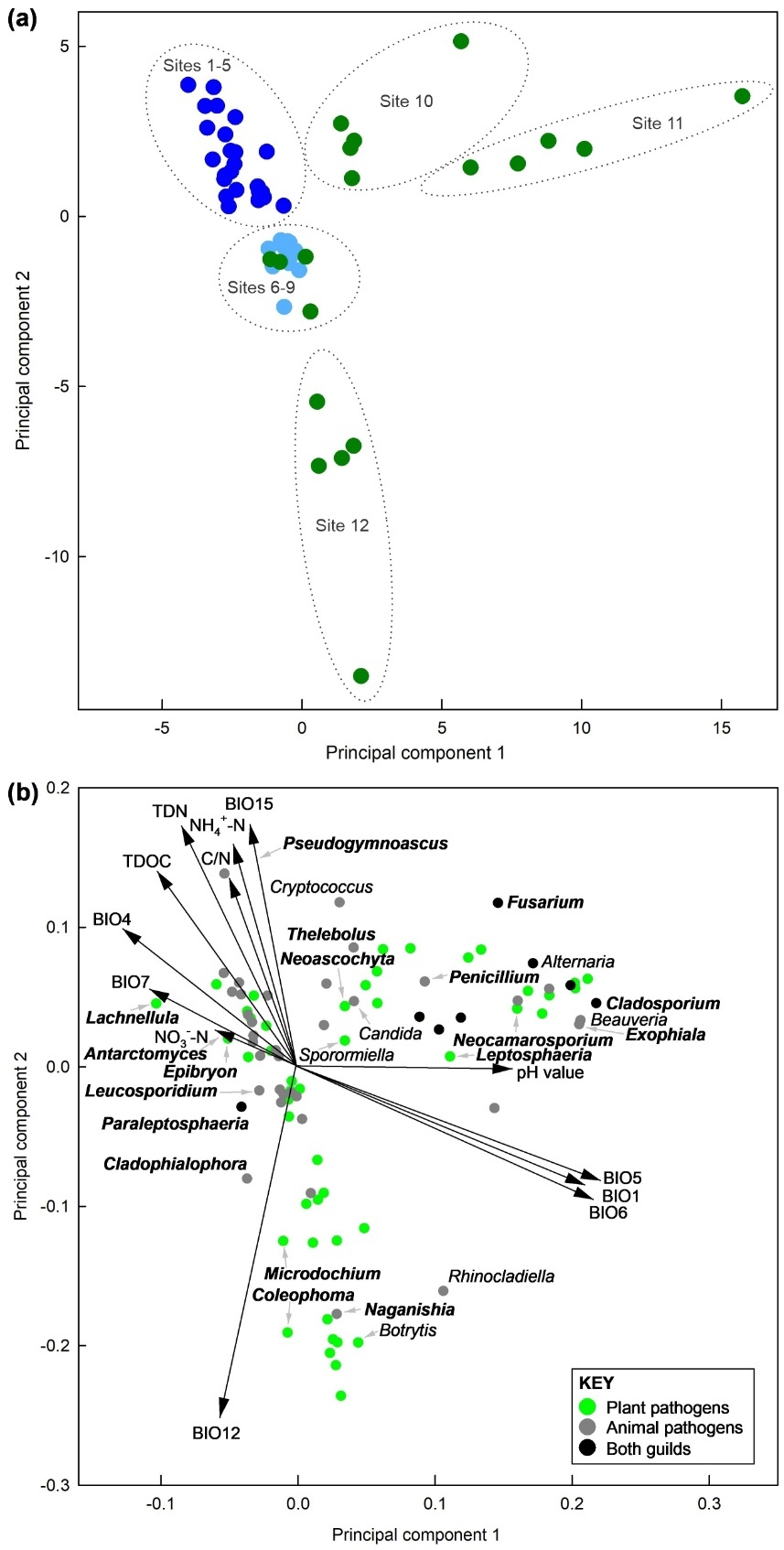


**Figure S7** | **Principal component analysis of associations between climatic and edaphic factors and the abundances of pathogenic fungal genera.** (a) Score plot. Ellipses denote individual sites or groups of sites. Sampling sites are colour-coded for geographical region (see key in Figure S2). (b) Loading plot. Black arrows denote vectors for climatic and edaphic factors and light green, grey and black circles denote genera of plant pathogens, animal pathogens and genera in both guilds, respectively (see key). The 10 most relatively abundant plant and animal pathogenic genera (including two genera assigned to both guilds) are labelled in bold italics. Seven other genera indicative of climatic factors (see Figures 5 and 6) are labelled in plain italics. Note that the genera recorded, which consist predominantly of ascomycetes and basidiomycete yeasts, are broadly representative of the fungi inhabiting barren Antarctic soils (Pointing et al. 2009; Newsham et al. 2021).


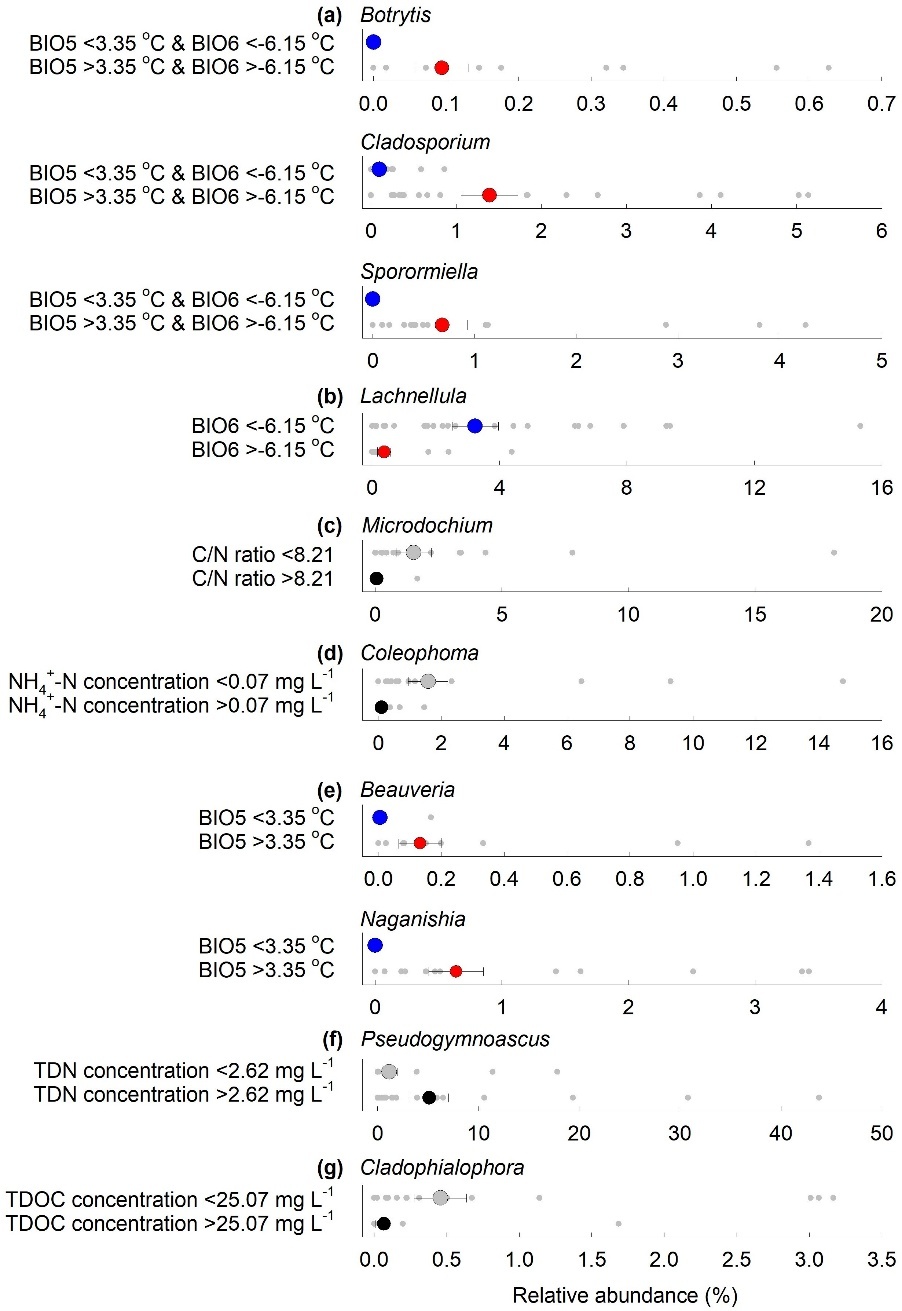


**Figure S8 |** **Soilborne plant and animal pathogenic fungal genera indicative of climatic and edaphic factors.** (a) Mean relative abundances of *Botrytis*, *Cladosporium* and *Sporormiella* in soils at sites with BIO5 values of <3.35 °C and >3.35 °C, and BIO6 values of <-6.15 °C and >-6.15 °C, represented by blue and red circles, respectively. (b) Mean relative abundances of *Lachnellula* at sites with BIO6 values of <-6.15 °C and >-6.15 °C, represented by blue and red circles, respectively. (c) Mean relative abundances of *Microdochium* in soils with C/N ratios of <8.21 and >8.21, represented by grey and black circles, respectively. (d) Mean relative abundances of *Coleophoma* in soils with NH_4_^+^-N concentrations of <0.07 mg L^-1^ and >0.07 mg L^-1^, represented by grey and black circles, respectively. (e) Mean relative abundances of *Beauveria* and *Naganishia* at sites with BIO5 values of <3.35 °C and >3.35 °C, represented by blue and red circles, respectively. (f) Mean relative abundances of *Pseudogymnoascus* in soils with total dissolved nitrogen concentrations of <2.62 mg L^-1^ and >2.62 mg L^-1^, represented by grey and black circles, respectively. (g) Mean relative abundances of *Cladophialophora* in soils with total dissolved organic carbon concentrations of <25.07 mg L^-1^ and >25.07 mg L^-1^, represented by grey and black circles, respectively. Values are means ± SEM and grey dots show individual data points. *Abbreviations*: TDN, total dissolved nitrogen; TDOC, total dissolved organic carbon. Pairwise comparisons in panels (a), (b), (e) and (f) were significant at *P*<0.05 following Benjamini-Hochberg correction. Those in panels (c), (d) and (g) were significant at *P*<0.07 following Benjamini-Hochberg correction.

**Appendix 1**

**R script used to retrieve and analyse data**

################################################################

###Data retrieval from CHELSA#####################################

################################################################

#download tif files (one for each bioclimatic variable)

#for reference, here are the links to the CHELSA files: https://envicloud.wsl.ch/#/?prefix=chelsa%2Fchelsa_V2%2FGLOBAL%2Fclimatologies%2F

#to download the files of interest, type the following commands in the terminal

#wget https://os.zhdk.cloud.switch.ch/envicloud/chelsa/chelsa_V2/GLOBAL/climatologies/1981-2010/bio/CHELSA_bio1_1981-2010_V.2.1.tif

#wget https://os.zhdk.cloud.switch.ch/envicloud/chelsa/chelsa_V2/GLOBAL/climatologies/1981-2010/bio/CHELSA_bio4_1981-2010_V.2.1.tif

#wget https://os.zhdk.cloud.switch.ch/envicloud/chelsa/chelsa_V2/GLOBAL/climatologies/1981-2010/bio/CHELSA_bio5_1981-2010_V.2.1.tif

#wget https://os.zhdk.cloud.switch.ch/envicloud/chelsa/chelsa_V2/GLOBAL/climatologies/1981-2010/bio/CHELSA_bio6_1981-2010_V.2.1.tif

#wget https://os.zhdk.cloud.switch.ch/envicloud/chelsa/chelsa_V2/GLOBAL/climatologies/1981-2010/bio/CHELSA_bio7_1981-2010_V.2.1.tif

#wget https://os.zhdk.cloud.switch.ch/envicloud/chelsa/chelsa_V2/GLOBAL/climatologies/1981-2010/bio/CHELSA_bio12_1981-2010_V.2.1.tif

#wget https://os.zhdk.cloud.switch.ch/envicloud/chelsa/chelsa_V2/GLOBAL/climatologies/1981-2010/bio/CHELSA_bio15_1981-2010_V.2.1.tif

#set working directory

setwd("~/Desktop/Dataset_prj")

#import raster objects

library(terra)

ras_bio1 <- rast("CHELSA_bio1_1981-2010_V.2.1.tif")

ras_bio4 <- rast("CHELSA_bio4_1981-2010_V.2.1.tif")

ras_bio5 <- rast("CHELSA_bio5_1981-2010_V.2.1.tif")

ras_bio6 <- rast("CHELSA_bio6_1981-2010_V.2.1.tif")

ras_bio7 <- rast("CHELSA_bio7_1981-2010_V.2.1.tif")

ras_bio12 <- rast("CHELSA_bio12_1981-2010_V.2.1.tif")

ras_bio15 <- rast("CHELSA_bio15_1981-2010_V.2.1.tif")

#import the geographical coordinates

pointCoordinates <- data.frame(read.csv("geo_data.csv", row.names = 1))

dim(pointCoordinates)

#[1] 12 2

#extract values from raster for the geographical coordinates of interest

rasValue_bio1 = extract(ras_bio1, pointCoordinates[,c(2,1)])[,2] #column 2 reports longitude and column 1 reports latitude

rasValue_bio4 = extract(ras_bio4, pointCoordinates[,c(2,1)])[,2] #column 2 reports longitude and column 1 reports latitude

rasValue_bio5 = extract(ras_bio5, pointCoordinates[,c(2,1)])[,2] #column 2 reports longitude and column 1 reports latitude

rasValue_bio6 = extract(ras_bio6, pointCoordinates[,c(2,1)])[,2] #column 2 reports longitude and column 1 reports latitude

rasValue_bio7 = extract(ras_bio7, pointCoordinates[,c(2,1)])[,2] #column 2 reports longitude and column 1 reports latitude

rasValue_bio12 = extract(ras_bio12, pointCoordinates[,c(2,1)])[,2] #column 2 reports longitude and column 1 reports latitude

rasValue_bio15 = extract(ras_bio15, pointCoordinates[,c(2,1)])[,2] #column 2 reports longitude and column 1 reports latitude

#merge and save data in .csv file

bioclimatic_variables=cbind(pointCoordinates, rasValue_bio1,rasValue_bio4,rasValue_bio5,

rasValue_bio6,rasValue_bio7,rasValue_bio12,rasValue_bio15)

dim(bioclimatic_variables)

#[1] 12 12

write.csv(bioclimatic_variables, "bioclimatic_variables.csv")

################################################################

###Data retrieval from CHELSA-BIOCLIM+###########################

################################################################

#download tif files (one for each bioclimatic variable, ssp, time period and model)

#for reference, here is the link to the CHELSA files: https://envicloud.wsl.ch/#/?prefix=chelsa%2Fchelsa_V2%2FGLOBAL%2Fclimatologies%2F

#to download the files of interest look into envidatS3paths.txt to see web links

#set working directory

setwd("~/Desktop/Dataset_prj")

#import libraries

library(terra)

#import the geographical coordinates

pointCoordinates <- data.frame(read.csv("geo_data.csv", row.names = 1))

dim(pointCoordinates)

#[1] 12 2

#define parameters

time_periods <- c("2011-2040", "2041-2070", "2071-2100")

ssps <- c("ssp126", "ssp370", "ssp585")

bio_vars <- c(1, 5, 6, 12)

models <- c("gfdl-esm4", "ipsl-cm6a-lr", "mpi-esm1-2-hr", "mri-esm2-0", "ukesm1-0-ll")

#start looping

for (model in models) {

#list to accumulate all data per model

all_model_data <- list()

for (period in time_periods) {

for (ssp in ssps) {

extracted_values <- list()

for (bio in bio_vars) {

#build filename and full path (adjust path if needed)

file_name <- sprintf("CHELSA_bio%d_%s_%s_%s_V.2.1.tif", bio, period, model, ssp)

file_path <- file.path(model, file_name)

if (!file.exists(file_path)) {

warning(paste("File not found:", file_path))

next

}

ras <- rast(file_path)

val <- extract(ras, pointCoordinates[, c(2, 1)])[, 2]

extracted_values[[paste0("bio", bio)]] <- val

}

if (length(extracted_values) > 0) {

#combine point coordinates + extracted raster values

bioclim_data <- cbind(pointCoordinates, as.data.frame(extracted_values))

#add columns to identify period and ssp

bioclim_data$period <- period

bioclim_data$ssp <- ssp

bioclim_data$sample <- row.names(pointCoordinates)

#store in list

all_model_data[[paste(period, ssp, sep = "_")]] <- bioclim_data

}

}

}

#combine all period-ssp data for this model into one dataframe

if (length(all_model_data) > 0) {

combined_data <- do.call(rbind, all_model_data)

#save to one CSV per model

write.csv(combined_data, paste0("bioclim_", model, ".csv"), row.names = FALSE)

cat("Saved combined file for model:", model, "\n")

} else {

warning(paste("No data extracted for model", model))

}

}

################################################################

###LASSO regressions#############################################

################################################################

#Note that the code below was used to analyse four datasets, *viz*. the richness of plant pathogens (filename 'div_plant_pathogens_soil_rarefied.csv'), the Hellinger-transformed relative abundance of plant pathogens (filename 'abund_plant_pathogens_soil_Hell.csv'), the richness of animal pathogens (filename 'div_animal_pathogens_soil_rarefied.csv') and the Hellinger-transformed relative abundance of animal pathogens (filename 'abund_animal_pathogens_soil_Hell.csv'), respectively. Input data are shown in Newsham *et al*. (2025), *richness and abundance of plant and animal pathogenic fungi in Patagonian, sub-Antarctic and Maritime Antarctic soil samples collected January–February 2018 (Version 1.0)* (https://doi.org/10.5285/d93d9ae6-4eff-4d9a-9997-d0ed06c3df1b) in the ‘data for analyses.csv’ file.

library(tidyverse)

data = read_csv('div_plant_pathogens_soil_rarefied.csv')

y=data$No._taxa

data = dplyr::select(data, -No._taxa)

library(glmnet)

n = length(y)

fit <- glmnet(data,y,family="gaussian",intercept=T,standardize=T,alpha=1)

preds <- predict(fit,newx=as.matrix(data))

coefs_chosen <- fit$beta[,33]

preds_chosen <- preds[,33]

fit$lambda

coef(fit)

preds_chosen

data_with_y = data

data_with_y$Y = y

lm_full <- lm(Y ~ BIO1 + BIO5 + pH + NH4, data = data_with_y)

anova(lm_full)

library(tidyverse)

data = read_csv('abund_plant_pathogens_soil_Hell.csv')

y=data$abund

data = dplyr::select(data, -abund)

library(glmnet)

n = length(y)

fit <- glmnet(data,y,family="gaussian",intercept=T,standardize=T,alpha=1)

preds <- predict(fit,newx=as.matrix(data))

coefs_chosen <- fit$beta[,15]

preds_chosen <- preds[,15]

fit$lambda

coef(fit)

preds_chosen

data_with_y = data

data_with_y$Y = y

lm_full <- lm(Y ~ BIO1 + BIO6 + BIO12 + pH, data = data_with_y)

anova(lm_full)

library(tidyverse)

data = read_csv('div_animal_pathogens_soil_rarefied.csv')

y=data$No._taxa

data = dplyr::select(data, -No._taxa)

library(glmnet)

n = length(y)

fit <- glmnet(data,y,family="gaussian",intercept=T,standardize=T,alpha=1)

preds <- predict(fit,newx=as.matrix(data))

coefs_chosen <- fit$beta[,22]

preds_chosen <- preds[,22]

fit$lambda

coef(fit)

preds_chosen

data_with_y = data

data_with_y$Y = y

lm_full <- lm(Y ~ BIO1 + BIO5 + BIO12 + pH, data = data_with_y)

anova(lm_full)

library(tidyverse)

data = read_csv('abund_animal_pathogens_soil_Hell.csv')

y=data$abund

data = dplyr::select(data, -abund)

library(glmnet)

n = length(y)

fit <- glmnet(data,y,family="gaussian",intercept=T,standardize=T,alpha=1)

preds <- predict(fit,newx=as.matrix(data))

coefs_chosen <- fit$beta[,23]

preds_chosen <- preds[,23]

fit$lambda

coef(fit)

preds_chosen

data_with_y = data

data_with_y$Y = y

lm_full <- lm(Y ~ BIO5 + BIO12 + CN + NH4, data = data_with_y)

anova(lm_full)

################################################################

###Indicator analyses##############################################

################################################################

#Note that data were organized into samples (in rows) and genera (in columns), with (in the example below) the first 28 and 25 rows being samples with values below and above the median value for each climatic or edaphic factor, respectively. Input data are shown in Newsham *et al*. (2025), *richness and abundance of plant and animal pathogenic fungi in Patagonian, sub-Antarctic and Maritime Antarctic soil samples collected January–February 2018 (Version 1.0)* (https://doi.org/10.5285/d93d9ae6-4eff-4d9a-9997-d0ed06c3df1b) in the ‘data for analyses.csv’ file.

library(indicspecies)

# IMPORT DATA MANUALLY

View(filename.csv)

attach(filename.csv)

groups=c(rep(1,28),rep(2,25))

groups

indval = multipatt(filename.csv, groups, control = how(nperm=999))

summary(indval)
